# Evaluation of Tazemetostat as a Therapeutically Relevant Substance in Biliary Tract Cancer

**DOI:** 10.3390/cancers15051569

**Published:** 2023-03-02

**Authors:** Dino Bekric, Daniel Neureiter, Celina Ablinger, Heidemarie Dobias, Marlena Beyreis, Markus Ritter, Martin Jakab, Johannes Bischof, Ulrich Koller, Tobias Kiesslich, Christian Mayr

**Affiliations:** 1 Center of Physiology, Pathophysiology and Biophysics, Institute of Physiology and Pathophysiology, Paracelsus Medical University, 5020 Salzburg, Austria; 2 Institute of Pathology, Paracelsus Medical University/University Hospital Salzburg (SALK), 5020 Salzburg, Austria; 3 Cancer Cluster Salzburg, 5020 Salzburg, Austria; 4 Institute of Pharmacy, Paracelsus Medical University, 5020 Salzburg, Austria; 5 Ludwig Boltzmann Institute for Arthritis and Rehabilitation, Paracelsus Medical University, 5020 Salzburg, Austria; 6 Gastein Research Institute, Paracelsus Medical University, 5020 Salzburg, Austria; 7 Kathmandu Medical School of Medical Sciences, Dhulikhel 45200, Nepal; 8 Research Program for Molecular Therapy of Genodermatoses, EB House Austria, Department of Dermatology and Allergology, Paracelsus Medical University/University Hospital Salzburg (SALK), 5020 Salzburg, Austria; 9 Department of Internal Medicine I, Paracelsus Medical University/University Hospital Salzburg (SALK), 5020 Salzburg, Austria

**Keywords:** biliary tract cancer, cholangiocarcinoma, EZH2 inhibitor, epigenetics, tazemetostat

## Abstract

**Simple Summary:**

Treating biliary tract cancer (BTC) successfully remains to be a difficult task. Standard therapeutic options encompass surgery, radiation and chemotherapy, but the median survival has not improved beyond one year. The reasons for this might be diagnosis at an already late stage and resistance towards current therapy. Therefore, novel strategies to combat this gastrointestinal disease need to be investigated. One alternative option may be to inhibit the enhancer of Zeste homolog 2 (EZH2), a histone-lysine-N-methyltransferase that was already shown to play a role in oncogenesis in BTC. Tazemetostat, an FDA-approved EZH2-inhibitor, seems to harbor promising anti-cancer properties in various tumor types. Therefore, in this study, we aim to investigate for the first time if tazemetostat might be a potential novel therapeutic strategy in biliary tract cancer.

**Abstract:**

Biliary tract cancer (BTC) is a gastrointestinal malignancy associated with a poor survival rate. Current therapies encompass palliative and chemotherapeutic treatment as well as radiation therapy, which results in a median survival of only one year due to standard therapeutic ineffectiveness or resistance. Tazemetostat is an FDA-approved inhibitor of enhancer of Zeste homolog 2 (EZH2), a methyltransferase involved in BTC tumorigenesis via trimethylation of histone 3 at lysine 27 (H3K27me3), an epigenetic mark associated with silencing of tumor suppressor genes. Up to now, there are no data available regarding tazemetostat as a possible treatment option against BTC. Therefore, the aim of our study is a first-time investigation of tazemetostat as a potential anti-BTC substance in vitro. In this study, we demonstrate that tazemetostat affects cell viability and the clonogenic growth of BTC cells in a cell line-dependent manner. Furthermore, we found a strong epigenetic effect at low concentrations of tazemetostat, which was independent of the cytotoxic effect. We also observed in one BTC cell line that tazemetostat increases the mRNA levels and protein expression of the tumor suppressor gene Fructose-1,6-bisphosphatase 1 (FBP1). Interestingly, the observed cytotoxic and epigenetic effects were independent of the mutation status of EZH2. To conclude, our study shows that tazemetostat is a potential anti-tumorigenic substance in BTC with a strong epigenetic effect.

## 1. Introduction

Biliary tract cancer (BTC) is a dismal gastrointestinal disease with a very poor 5-year survival rate [1]. A possible explanation for the poor survival rate of BTC might be that symptoms are very unspecific, leading to a diagnosis at an already advanced stage [2]. For instance, typical symptoms of BTC are abdominal pain, unspecific weight loss and painless jaundice which impairs an efficient clinical management of BTC [3]. Current therapies against BTC encompass palliative treatment, radiation therapy and a combinatorial chemotherapeutic treatment, consisting of cisplatin and gemcitabine. However, the median survival remains poor [2,4]. Additionally, second-line therapies for advanced BTC are not standardized [5]. Due to the lack of efficient treatments as well as the poor overall survival rate, the investigation of new therapeutic approaches is still necessary.

Enhancer of Zeste homolog 2 (EZH2) is the catalytic subunit of the polycomb repressive complex 2 (PRC2), which is an epigenetic regulator, that specifically performs stepwise trimethylation of histone H3 at Lysine 27 (H3K27me3), using S-adenosyl methionine cofactor (SAM) as the methyl donor [6]. These methylations result in the formation of a heterochromatin complex and gene silencing [6]. Physiologically, EZH2 is involved in embryonic development by regulating the expression and maintenance of genes, of which are required for differentiation and development during the embryonic phase [6]. Besides EZH2, the PRC2 consists of the core components EED, LSD1, SUZ12, DNMT1 and JARID2, which are mandatory for the proper function of the PRC2 [6].

Besides its role in embryonic development, aberrant PRC2 and EZH2 activity has been described in several human cancer types. It was demonstrated that EZH2 is overexpressed and/or harbors a gain-of-function mutations in solid tumors such as breast and prostate cancer as well in lymphomas and that these changes in EZH2 function are associated with shorter overall survival, progression of disease with development of metastasis and a higher TNM stage [7,8,9,10].

In BTC, EZH2 was also shown to be overexpressed [8,11]. Liu et al. demonstrated via immunostaining that patients with higher EZH2 expression suffered from larger tumors, more frequent lymph node metastases and a poorer overall survival compared to patients with a lower or negative EZH2 expression [12]. Additionally, Sasaki et al. and Liu et al. demonstrated in BTC that on a molecular level, EZH2 expression was negatively correlated with the expression of the tumor suppressor genes PTEN and p16, whereas Yamaguchi et al. found that the Ki-67, as a marker of proliferation, was positively correlated with EZH2 expression [11,13,14]. Tang et al. could demonstrate that EZH2 was highly expressed in cholangiocarcinoma (CCA) cells and that the overexpression of EZH2 led to the inhibition of apoptosis and resulted in an elevated proliferation of CCA cells [15].

Furthermore, the study by Tang et al. showed that RUNX3, a well-known tumor suppressor, was downregulated by the EZH2-mediated methylation of H3K27. Additionally, EZH2 inhibition resulted in upregulated RUNX3 protein expression, induced apoptosis and reduced cell proliferation [15].

In another study, carried out by Zhang et al., it was shown that in a xenograft model, EZH2 knockdown was able to reduce the progression of CCA significantly, and the depletion of EZH2 in CCA cells reduced the colony and growth formation ability [16].

Therefore, EZH2 may represent an attractive target for pharmacological interventions.

Tazemetostat (also known as E7438 or EPZ-6438) is a SAM competitive EZH2 inhibitor that is currently used in more than 40 clinical trials in different clinical settings [17], (https://clinicaltrials.gov/ct2/results?cond=&term=TAZEMETOSTAT&cntry=&state=&city=&dist=, accessed on 19 September 2022). On January 2020, the FDA approved tazemetostat (Tazverik) for locally advanced or metastatic epithelioid sarcoma that are not eligible for complete surgical removal [18]. In several other studies, the anti-tumorigenic properties of tazemetostat were demonstrated: For example, Zhou et al. demonstrated that tazemetostat was able to sensitize mouse oral squamous cell carcinoma model cells (MOC-esc1) to T-cell-mediated cytotoxicity [19]. Furthermore, tazemetostat was able to increase cytotoxicity in head and neck cancer cells compared to untreated cells by enhancing the antigen presentation of tumor cells. Likewise, Tan et al. observed an augmentation of the cytotoxic effect of the chemotherapeutic 5-Flourouracil (5-FU) in colorectal cancer when combined with tazemetostat [20]. In medulloblastoma, Zhang et al. could demonstrate that the inhibition of EZH2 by tazemetostat led to the activation of the tumor suppressor gene ADGRB1, which resulted in an anti-tumorigenic response [21]. Additionally, SAM competitors such as tazemetostat worked more efficiently in cells harboring a gain-of-function mutation in EZH2 at lysine at position 641/646, which is positioned in the SET domain of EZH2 [22]. In 2014, Knutson et al. demonstrated that non-Hodgkin lymphoma (NHL) cells which displayed an EZH2 point mutation were more susceptible towards tazemetostat than wild-type EZH2 cells [22]. Almost all used NHL cells that harbored a Y646 mutation of EZH2 displayed higher sensitivity towards tazemetostat compared to wild-type cells [22]. Interestingly, cell proliferation was inhibited via apoptosis induction and cell cycle arrest in EZH2-mutant lymphoma cells if tazemetostat was applied [22].

Based on the current literature, the EZH2 inhibitor tazemetostat harbors potential as an (adjuvant) anti-tumor drug. The involvement of EZH2 in BTC development and progression is well described. However, data regarding tazemetostat and BTC are missing. Therefore, our presented study aims to investigate the cytotoxic and epigenetic effects of tazemetostat using an in vitro model with different human BTC cells for the first time.

## 2. Materials and Methods

### 2.1. Cell Culture and Substances

Human BTC cell lines HuCCT1 (JCRB0425, [23]), KKU-055 (JCRB1551), NOZ (JCRB1033, [24]), OCUG-1 (JCRB0191, [25]) and OZ (JCRB1032, [26]) and non-cholangiocyte cell line MMNK-1 (JCRB1553) were purchased from the Japanese Collection of Research Bioresources Cell Bank (JCRB, Osaka, Japan). BTC cell lines (Human) EGI-1 (ACC-385, [27]) and TFK-1 (ACC-344, [28]) were purchased from the German Quotes from Collection of Microorganisms and Cell Culture (DSZM, Braunschweig, Germany). Cell lines were cultured in a humidified atmosphere (5% CO_2_, 37 °C) in Dulbecco’s modified Eagle’s medium with high glucose (DMEM; Gibco, ThermoFisher Scientific, Vienna, Austria), supplemented with 10% fetal bovine serum (FBS; Eximus, Catus Biotech, Germany), 1% antibiotic-antimycotic (Sigma-Aldrich, St. Louis, MO, USA), 10 mM HEPES (Pan Biotech, Aidenbach, Germany) and 1 mM sodium pyruvate (Pan Biotech). Dulbecco’s Phosphate Buffered Saline (DPBS; Pan Biotech) was used for washing steps. Cell harvesting was carried out with 0.25% trypsin-EDTA (Sigma-Aldrich). Cells were counted using a Spark multimode reader and Cell Counting Chips (Tecan, Grödig, Austria).

Resazurin was purchased from Alfa Aesar (Kandel, Germany) and dissolved in DPBS. Cisplatin, purchased from Selleckchem (Houston, TX, USA), was dissolved in ddH_2_O to a stock concentration of 5 mM and stored in aliquots at −20 °C. Tazemetostat was purchased from Selleckchem, was dissolved in dimethyl sulfoxide (DMSO; Sigma Aldrich) to a stock concentration of 20 mM and stored in aliquots at −20 °C. Samples treated with solvent did not significantly differ from untreated samples.

### 2.2. Clonogenic Assay

Optimal cell densities for a miniaturized clonogenic assay in 96-well microplates (Starlab, Hamburg, Germany) were determined as described [29]. The following seeding numbers per well were chosen: 80 cells for HuCCT1 and OCUG-1, 50 cells for KKU-055, 40 cells for EGI-1, and 30 cells for NOZ and the MMNK-1 cells. The seeding of OZ and TFK-1 cells at different cell numbers did not result in clonogenic growth. Therefore, these cell lines were excluded from the experiments. The determination of optimal cell density was carried out in biological and technical triplicates.

For the investigation of clonogenic growth, cells were seeded according to the determined optimal seeding numbers in 96-well microplates and were grown overnight. Then, cells were washed with DMEM without serum and incubated with different concentrations of tazemetostat in DMEM with serum using a 1:2 dilution series (starting concentration 80 µM, 10 steps) for seven days. To avoid evaporation, empty spaces on the plate were filled with DPBS. Confluence was measured after seven days with the Spark multimode reader.

### 2.3. Cell Viability Analysis

The short-term cytotoxicity of tazemetostat was measured after 72 and 120 h of tazemetostat treatment using the resazurin assay. Cells were seeded in 96-well microplates (10,000 cells per well for 72 h time point; 6000 cells for 120 h time point) and let to grow overnight. Then, cells were washed with serum-free DMEM and incubated with tazemetostat in FBS-free DMEM using a serial dilution (starting from 100 µM, 1:2, 10 steps). After 72 h or 120 h incubation, respectively, resazurin was added and fluorescence was measured on a Spark multimode reader.

Serum-free medium was used to avoid the interactions of serum components with tazemetostat.

Based on Knutson et al., the long-term cytotoxic effects of tazemetostat (up to 360 h incubation time) were investigated as followed: 6000 cells for KKU-055 and 4000 cells for NOZ were seeded in a 96-well microplate and let to grow overnight [22]. Then, cells were washed with FBS-free DMEM and incubated with 0.3, 3 and 30 µM Tazemetostat, respectively. After 120 h of incubation time, resazurin was added to the selected wells for measurement of cell viability. For the remaining wells, cells were harvested with trypsin-EDTA, pooled (for each condition), counted, re-seeded at the described seeding densities and let to grow overnight without tazemetostat. Cells were again washed and then incubated with tazemetostat (0.3, 3 and 30 µM) for an additional 120 h to evaluate the viability after 240 h. The procedure was repeated an additional time to measure cell viability also after 360 h.

### 2.4. Western Blot

KKU-055, NOZ and OCUG-1 were seeded in 60 mm dishes at a seeding density of 5.2 × 10^6^ per dish and let to grow overnight. Cells were washed with FBS-free DMEM, incubated with 0.3 µM tazemetostat for 96 h, washed with DPBS, harvested with trypsin-EDTA, centrifuged, counted and stored as cell pellets at −20 °C. For protein expression analysis, pellets were thawed, DPBS was added to obtain a concentration of 10^7^ cells per ml and cells were lysed via sonication with a Sonopuls HD70 (UW 70 ultrasound head, Bandelin; 10 pulses). Samples were then centrifuged (17,000× *g*, 10 min) and 10 µL of supernatant was mixed with 10 µL of 2× sodium dodecyl sulfate (SDS) containing a lysis buffer (SDS; Thermo Fisher Scientific, Waltham, MA, USA), incubated for 5 min at 95 °C and centrifuged again (400× *g*, 5 min at RT). Proteins, with each slot containing 200,000 cells, were separated on gradient SDS gels (20 µL of each sample; 4–20% Mini-PROTEAN gels, Biorad, Hercules, CA, USA) for 90 min at 100 V and transferred using a Trans-Blot^®^ Turbo™ System and nitrocellulose membranes (Biorad). Unspecific binding was blocked using a Blotting-Grade Blocker (Biorad). Membranes were incubated overnight at 4 °C with primary antibodies: anti-H3 (1:2000), anti-H3K27me3 (1:1000), anti-FBP1 (1:1000) and anti-EZH2 (1:1000)—all diluted in Blotting-Grade Blocker and purchased from Cell Signaling Technology (Danvers, MA, USA). Blots were washed three times with TBS-T, incubated with the secondary antibody (anti-rabbit IgG HRP-linked, 1:1000, Cell Signaling Technology) for 90 min at room temperature and then incubated for 2 min with the Signal Fire ECL Reagent (Cell Signaling Technologies) for signal development. Chemiluminescence was analyzed with the ChemiDoc MP System and the Image Lab Software™ (Biorad). Grey densities of bands were calculated with ImageJ (V1.53, NIH, Bethesda, MD, USA) to evaluate the protein expression related to loading control H3. Fold regulation, the negative inverse of fold change, was calculated to demonstrate the up- or downregulation of genes.

### 2.5. Real-Time PCR

The BTC cell lines KKU-055 and NOZ were seeded in 60 mm dishes at a seeding density of 5.2 × 10^6^ per dish, grown overnight, washed with FBS-free DMEM and incubated with 0.3 µM tazemetostat for 96 h, respectively. Total RNA was isolated with TRI Reagent (Merck, Rahway, NJ, USA) and a Direct-zol RNA Miniprep kit (Zymo Research, Irvine, CA, USA) according to the manufacturers’ instructions. cDNA synthesis was carried out using the GoScript™Reverse Transcriptase kit (Promega, Madison, WI, USA). Real Time PCR was performed with a ViiA7 real-time PCR system (Applied Biosystems, Thermo Fisher Scientific) using the GoTaq^®^ Master Mix (SYBR^®^ Green, Promega). mRNA expression levels were related to ß-actin (ΔCt). Changes in mRNA expression between treated and untreated samples were calculated according to the ΔΔCt method. Fold regulation, the negative inverse of fold change, was calculated to demonstrate the up- or downregulation of genes. All primers were purchased from Sigma Aldrich (KiCqStart^®^ SYBR^®^ Green Primers) and prepared as 100 µM stocks (in H_2_O)—sequences are listed in Supplementary Figure S1.

### 2.6. Point Mutation Analysis

EGI-1, KKU-055, NOZ, OZ, TFK-1, HuCCT1, MMNK-1 and OCUG-1 were seeded in 60 mm dishes using a seeding density of 5.2 × 10^6^ per dish, let to grow overnight, harvested with trypsin-EDTA and centrifuged. Genomic DNA was extracted using a Wizard Genomic DNA Purification Kit (Promega), according to the manufacturer’s protocol. Concentration and quality of extracted DNA was measured with an Eppendorf Biophotometer^®^ plus (Hamburg, Germany) and amplification of the region of interest was carried out using GoTaq HotStart Polymerase (Promega) and specific primers (see Supplementary Figure S2) on a Thermocycler Labcycler^®^ Sensoquest (Göttingen, Germany). The PCR product was evaluated via gel electrophoresis and subsequently sequenced using Sanger sequencing. Evaluations of the sequenced files were carried out via Finch TV (v1.5, NIH, Geospiza, Inc.; Seattle, WA, USA).

### 2.7. Combination of Cisplatin and Tazemetostat

To investigate the possible synergistic cytotoxic effects of tazemetostat with the standard chemotherapeutic cisplatin, KKU-055 and NOZ cells were seeded with a seeding density of 5000 (KKU-055) and 10,000 (NOZ) in 96-well microplates. For the simultaneous treatment of cells with cisplatin and tazemetostat, cells were grown overnight, washed with serum-free DMEM and incubated with a sub-lethal concentration of tazemetostat (30 µM) and a cisplatin dilution series (1:2, 10 steps, highest concentration of 20 µM) for 72 h. Cell viability was then measured via the resazurin assay. In a second approach, cells were seeded as described and pre-treated with 30 µM tazemetostat for 96 h. Afterwards, cells were washed with FBS-free DMEM and incubated with a cisplatin dilution series (10-fold 1:2, highest concentration of 30 µM) without tazemetostat for an additional 72 h before the measurement of cell viability.

### 2.8. Immunohistochemistry

The three human BTC cell lines KKU-055, NOZ and OCUG-1 were seeded in 60 mm dishes and let to grow overnight. Cell blocks were prepared using a 1:1 mix of citrate plasma and Thromborel S (Siemens Healthcare, Marburg, Germany).

The prepared cell blocks of these BTC cell lines were immunohistochemically stained for CK7, EZH2 and Vimentin (see details of the used antibodies in Table 1). In brief, 4 µm sections were mounted on glass slides, deparaffinized using graded alcohols, subjected to antigen retrieval at pH 9 and stained using the primary antibodies listed below. Ultraview (Ventana, Oro Valley, AZ, USA) was used as an IHC detection kit.

### 2.9. Statistics and In Silico Analysis

If not stated otherwise, all data points represent the mean values of at least three independent biological replicates ± SEM, where each biological replicate contained an appropriate number of technical replicates. The Student’s *t*-test as well as ANOVA test with Bonferroni correction were applied for the calculation of significances between control and treated samples. All calculations were performed using OriginPro 9.1 (OriginLab, Northampton, MA, USA). Statistical results were considered significant (*) or highly significant (**) at *p* < 0.05 and *p* < 0.01, respectively.

The available biodata of EZH2 and FBP1 mRNA expression in human BTC samples were analyzed via GEPIA http://gepia.cancer-pku.cn, (accessed on 26 January 2023) [30]. DNA methylation status as well as the clinical significance of methylated FBP1 in BTC human samples were analyzed via DNMIVD http://www.unimd.org/dnmivd/ and the SMART App http:// http://www.bioinfo-zs.com/smartapp/, (accessed on 26 January 2023).

## 3. Results

### 3.1. Tazemetostat Affects Growth of BTC Cells

**Figure 1:**
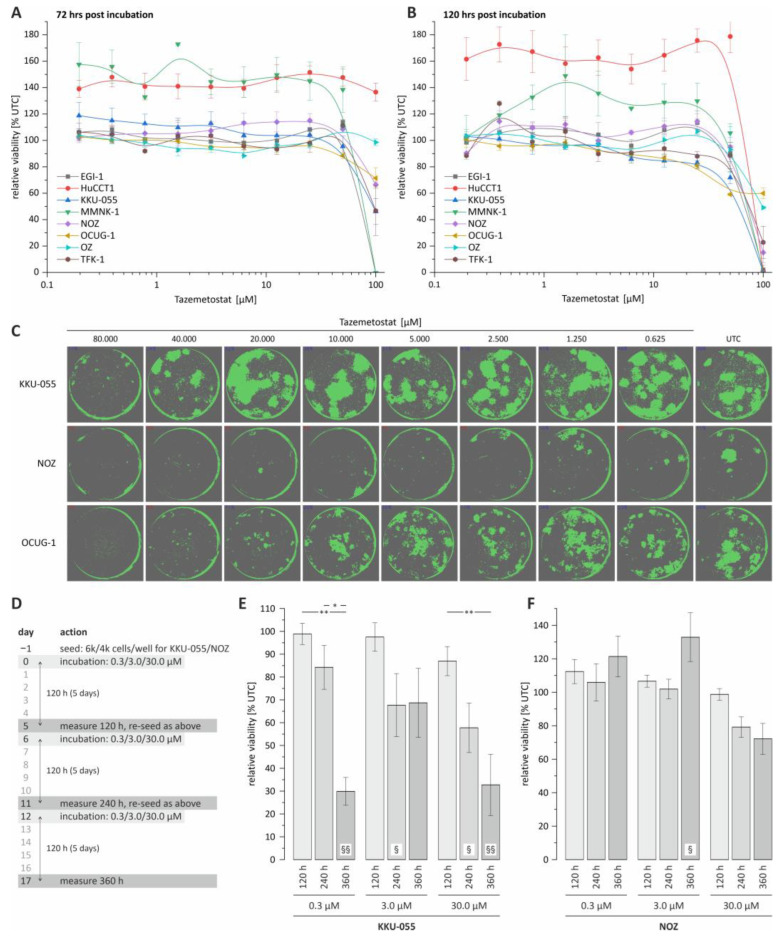
Cell viability and colony formation analysis of BTC cell lines following tazemetostat treatment. (**A**) Cell viability of 8 BTC cell lines following tazemetostat treatment for 72 h; (**B**) cell viability of 8 BTC cell lines following tazemetostat treatment for 120 h; (**C**) representative images of colony formation after treatment with tazemetostat for 7 d (KKU-055, NOZ and OCUG-1); (**D**) protocol of performing cell viability analysis for up to 360 h with tazemetostat treatment; (**E**) cell viability analysis of KKU-055 after 120 h, 240 h and 360 h with 0.3, 3 and 30 µM tazemetostat; (**F**) cell viability analysis of NOZ after 120 h, 240 h and 360 h with 0.3, 3 and 30 µM tazemetostat. * = significant *p* < 0.05; ** highly significant *p* < 0.01 between different time points of the same concentration. § = significant *p* < 0.05, §§ = highly significant *p* < 0.01 between selected time point and the untreated control of the same concentration; UTC = untreated control.

In the first step, we investigated the effect of tazemetostat on the viability of BTC cells following 72 and 120 h of treatment, respectively. As shown in Figure 1A,B, tazemetostat reduced the viability of most cell lines only at a very high concentration (starting from a concentration of 50 to 100 µM). We additionally investigated the effect of different tazemetostat concentrations on the clonogenic growth of BTC cells as an in vitro surrogate marker of the tumorigenic potential of cancer cells. We found that tazemetostat reduces clonogenic growth in a cell line-dependent manner. Figure 1C shows confluence images of KKU-055, OCUG-1 and NOZ cells as representative cell lines for a minor, moderate or strong effect of tazemetostat on clonogenic growth (see Supplementary Figure S3 for EGI-1, HuCCT-1 and MMNK-1 cells; due to their specific growth patterns, OZ and TFK-1 cells were not suitable for assessment of clonogenic growth). The strongest effect of tazemetostat on clonogenic growth was observable in NOZ, where at concentrations ≥2.5 µM, clonogenic growth was almost completely inhibited. In contrast, in KKU-055 cells, only treatment with high concentrations (≥40 µM) of tazemetostat resulted in a reduction in clonogenic growth. Regarding OCUG-1, a reduction in clonogenic growth was visible at concentrations of tazemetostat of ≥10 µM.

Based on the results of the clonogenic growth assay, we selected KKU-055 and NOZ cells for further experiments, as these cell lines are representative for cell lines with low and high sensitivity towards treatment with tazemetostat, respectively.

The current literature suggests a potential latency of the cytotoxic effect of tazemetostat in cancer cells [22]. Therefore, in an additional approach, we expanded the total incubation time to 360 h and measured the cell viability of the selected BTC cell lines after 120, 240 and 360 h of incubation with tazemetostat, respectively (see Figure 1D). Interestingly, for KKU-055 cells, which only showed a reduction in clonogenic growth at high tazemetostat concentrations, we measured a significant reduction in cell viability after 360 h of incubation time, even with the lowest tazemetostat concentration (0.3 µM, see Figure 1E). In contrast, in NOZ cells, only treatment with 30 µM tazemetostat for 240 and 360 h resulted in a non-significant reduction in cell viability (240 and 360 h, Figure 1F).

We also tested whether the co-treatment of BTC cells with tazemetostat and cisplatin leads to a synergistic cytotoxic effect. However, we found that neither the simultaneous treatment nor pre-incubation of cells with tazemetostat followed by treatment with cisplatin resulted in synergistic effects (Supplementary Figure S4).

### 3.2. Tazemetostat Significantly Reduces H3K27me3 Levels in BTC Cells

**Figure 2:**
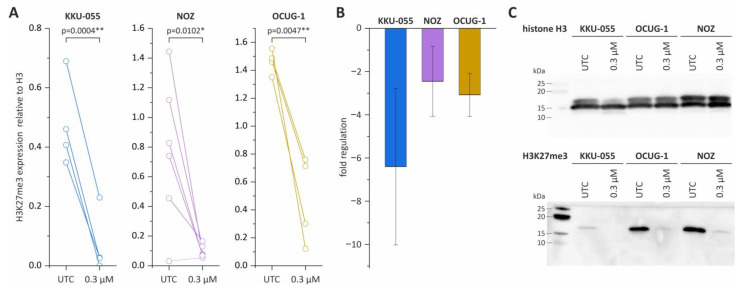
Western Blot analysis of H3K27me3 levels in KKU-055, NOZ and OCUG-1 following tazemetostat treatment. (**A**) The expression of H3K27me3 after tazemetostat treatment for 96 h in BTC cells; (**B**) changes in the fold regulation of H3K27me3 in BTC cell lines KKU-055, NOZ and OCUG-1 following tazemetostat treatment (96 h); (**C**) representative Western Blot images of H3 and H3K27me3 levels after incubation with tazemetostat for 96 h in BTC cell lines KKU-055, NOZ and OCUG-1. * = significant *p* < 0.05; ** highly significant *p* < 0.01, UTC = untreated control. The uncropped blot images are shown in Supplementary Figure S9.

Next, we investigated the epigenetic effect of tazemetostat on BTC cells and measured H3K27me3 levels. The treatment of KKU-055, NOZ and OCUG-1 cells with 0.3 µM tazemetostat resulted in a reproducible significant -2-fold to -6-fold reduction in H3K27me3 levels (Figure 2A–C).

### 3.3. EZH2 mRNA and Protein Expression Are Not Affected by Tazemetostat

**Figure 3:**
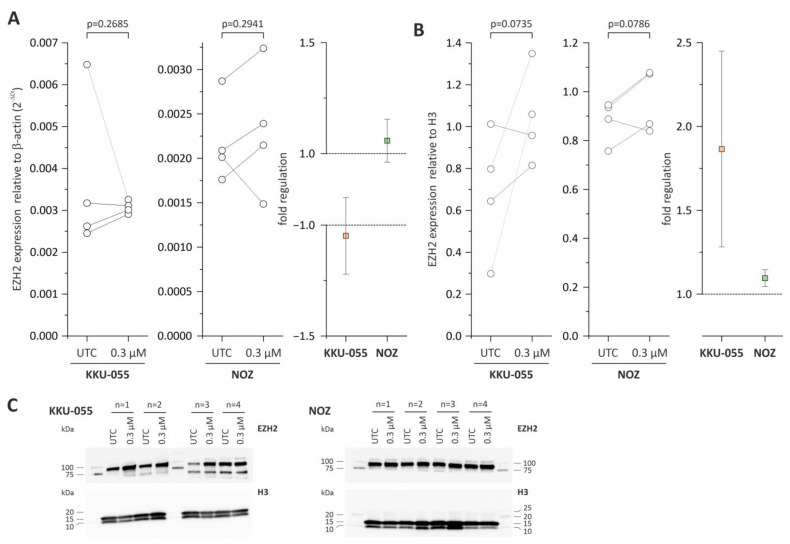
Western Blot and mRNA expression analysis of EZH2 in KKU-055 and NOZ following tazemetostat treatment. (**A**) EZH2 mRNA expression and fold regulation after incubation with tazemetostat for 96 h in KKU-055 and NOZ cells; (**B**) EZH2 protein expression and fold regulation after incubation with tazemetostat for 96 h in KKU-055 and NOZ cells; (**C**) representative Western Blot images of H3 and EZH2 after the incubation of tazemetostat for 96 h in BTC cell lines KKU-055 and NOZ. UTC = untreated control. The uncropped blots are shown in Supplementary Figure S9.

We next checked whether treatment with tazemetostat altered (compensatory) the EZH2 expression. As shown in Figure 3A, the mRNA levels of EZH2 were not changed by treatment with tazemetostat in both BTC cell lines. Similarly, on a protein level, no significant changes in EZH2 protein levels could be observed (Figure 3B,C).

### 3.4. FBP1 Is Upregulated in KKU-055 Cells after Treatment with Tazemetostat

**Figure 4:**
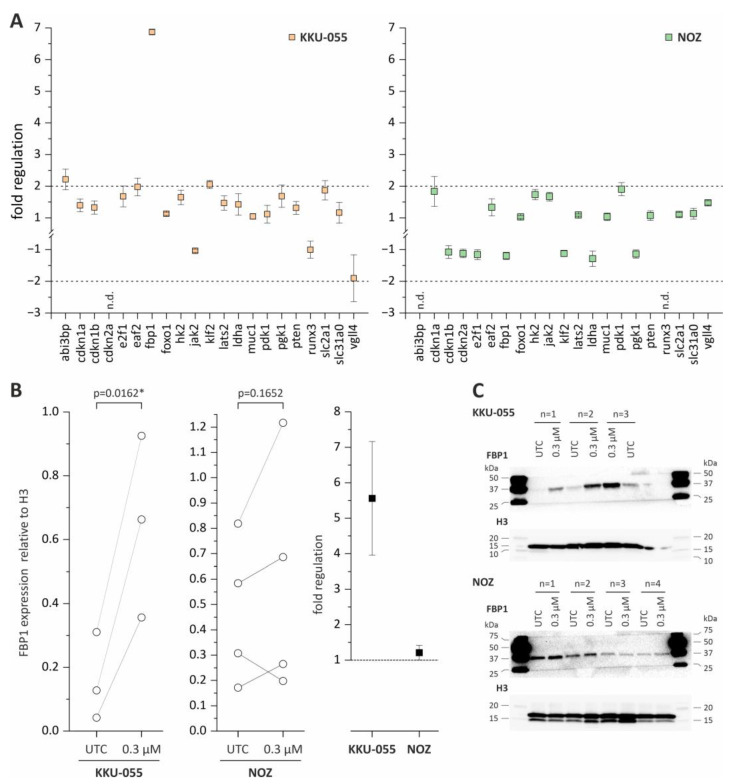
mRNA expression analysis of EZH2-associated genes and Western Blot analysis of FBP1 in KKU-055 and NOZ following tazemetostat treatment. (**A**) Fold regulation of 21 genes in KKU-055 and NOZ after treatment with tazemetostat for 96 h (+2 and −2 = significant change). (**B**) FBP1 protein expression and fold regulation after incubation with tazemetostat for 96 h in KKU-055 and NOZ cells. (**C**) Representative Western Blot images of H3 and FBP1 after incubation of tazemetostat for 96 h in BTC cell lines KKU-055, NOZ. * = significant *p* < 0.05, UTC = untreated control. The uncropped blots are shown in Supplementary Figure S9.

To investigate potential molecular mechanisms associated with the observed effects of tazemetostat in BTC cells, we measured the changes in mRNA levels of a total of 21 genes that were previously reported as directly regulated by EZH2 or part of molecular pathways that are regulated by EZH2. The selected genes, as well as their role in cancer and the references are listed in the Supplementary Figure S5. KKU-055 and NOZ cells were treated with 0.3 µM tazemetostat for 96 h before measurement of mRNA levels. Genes with a fold regulation of +2 and −2 were considered as upregulated and downregulated, respectively. As shown in Figure 4A, treatment with tazemetostat resulted in a significant 7-fold upregulation of the tumor suppressor fbp1 in KKU-055 cells. In addition, we also observed an increase (fold change > 2) of mRNA levels of klf2 and abi3bp in KKU-055 cells (Figure 4A). Of note, in NOZ cells, changes of mRNA levels of all 21 genes remained under the threshold of 2 (Figure 4A).

Since fbp1 mRNA levels were significantly enhanced in KKU-055 cells following tazemetostat treatment, we also measured protein levels of FBP1. In NOZ cells, the FBP1 protein expression was not affected following tazemetostat treatment, whereas in KKU-055, the FBP1 protein expression was significantly upregulated (Figure 4B,C).

Furthermore, when analyzing the available biodata of the Gene Expression Profiling Interactive Analysis (GEPIA) platform [30] (see http://gepia.cancer-pku.cn (accessed on 19 December 2022)) using data of The Cancer Genome Atlas (TCGA) project for cholangiocarcinoma, the mRNA expression of EZH2 and FBP1 in BTC samples showed a diametral distribution in normal controls and cases of BTCs, as demonstrated in Supplementary Figure S6: EZH2 was significantly upregulated in the tumor cases (Supplementary Figure S6A), whereas FBP1 was significantly downregulated in the tumor cases (Supplementary Figure S6A) and vice versa. Additionally, this expression pattern of EZH2 and FBP1 could be related, but not significantly, to the overall clinical outcome too, as shown in Supplementary Figure S6B, respectively. Furthermore, when analyzing the available biodata of the SMART app platform (http://www.bioinfo-zs.com/smartapp, accessed on 26 January 2023) and the DNMIVD (DNA Methylation Interactive Visualization Database, http://www.unimd.org/dnmivd/ (accessed on 26 January 2023)) database regarding the DNA methylation status of FBP1 in BTC human samples, it can be seen that in BTC tumor samples, the DNA methylation of FBP1 is higher compared to non-tumor samples (see Supplementary Figure S6C). Additionally, patients with a high DNA methylation at the FBP1 promotor region are suffering a non-significantly worse clinical outcome (see Supplementary Figure S6D).

### 3.5. BTC Cell Lines Harbor the EZH2 Gain-of-Function Mutation

According to previous studies, the effect of tazemetostat is dependent on the mutation status of EZH2 [22]. We, therefore, analyzed the mutation status of EZH2 in our BTC cell lines to investigate whether the observed cytotoxic and epigenetic effects of tazemetostat can be related to the mutation status of EZH2. As shown in Table 2, both KKU-055 and NOZ cells harbored no mutation in the EZH2 gene. However, we found a Y641S mutation in OCUG-1 and TFK-1 cells (see Supplementary Figure S7).

## 4. Discussion

In the current project, we provide first evidence that the FDA-approved EZH2 inhibitor tazemetostat possesses antitumor effects in BTC. We found that treatment with tazemetostat affected clonogenic growth in a cell line-dependent manner. Our data are in line with other findings regarding the effect of pharmacological EZH2 inhibition on clonogenic growth. Bate-Eya et al. could demonstrate that clonogenic growth was affected in neuroblastoma cell lines following tazemetostat treatment for 14 days [31]. Similar to our study, a reduction in clonogenic ability only occurred at much higher concentrations than needed for H3K27me3 reduction [31]. Interestingly, immunohistochemistry staining revealed that NOZ cell lines are epithelial, whereas KKU-055 and OCUG-1 display a mesenchymal phenotype, which might explain why the clonogenic ability of NOZ cells was more affected (see Supplementary Figure S8).

Regarding the effect of tazemetostat on cell viability, we found no significant changes after the application of tazemetostat for 72 h and for 120 h, as seen within other studies [22,32]. Therefore, we observed that clonogenic ability was affected by tazemetostat, whereas cell viability did not change at all after treatment with tazemetostat for up to 120 h.

However, other studies have pointed out that long-term incubation (>120 h) with tazemetostat is required since EZH2 inhibition, as an epigenetic intervention, has a certain latency before the manifestation of a reduction in cell viability by the delayed activation of specific tumor suppressor genes which are downstream targets of EZH2 [32,33]. For instance, Brach et al. could demonstrate that cytotoxic effects occurred only after treatment with tazemetostat for up to 240 h in diffuse large B-cell lymphomas [32]. Furthermore, Knutson et al. demonstrated that cell viability following tazemetostat treatment was reduced in NHL cells after 96 h of treatment, which might be explainable by the accompanied reduction in H3K27me3 levels in the same time frame [22]. In accordance with these studies, we found that a clear reduction in cell viability in selected BTC cell lines occurred only after 360 h of incubation with tazemetostat. However, several further investigations must be performed to clarify the possible underlying mechanisms of the heterogenic effect of tazemetostat in BTC cells, especially considering the mutational status of Y641. Furthermore, there is evidence that non-canonical PRC2s exist that contain the EZH2 homolog EZH1 as the histone methyltransferase [34]. The moderate effect of tazemetostat on the cell viability might be due to compensation of the inhibition of EZH2 by EZH1 [35]. For instance, Shinohara et al. could demonstrate, that in malignant rhabdoid tumor cells, EZH1 protein expression was upregulated, after tazemetostat treatment [36]. Additionally, lncRNAs as well as miRNAs, were also shown to interact with EZH2 in BTC, which might also be interesting for future investigations [37].

Although the effects of tazemetostat on clonogenic growth and cell viability were observable only at relative high concentrations, several studies demonstrated that the epigenetic effect occurs at significantly lower substance concentrations [22,31]. For example, in the study by Bate-Eya et al., relatively low nanomolar concentrations of tazemetostat were needed to reduce H3K27me3 levels significantly (62.5 nM), whereas clonogenic ability was impaired at relatively higher concentrations (1 µM) [31].

Likewise, in our study, in KKU-055 and NOZ cells, we were able to measure the effects of tazemetostat on cell growth only at concentrations in the (high) µM range and after long incubation times, whereas treatment with 0.3 µM tazemetostat resulted in a clear reduction in H3K27me3 levels after 96 h of treatment. This is in line with several other studies [22,32,38].

It is well established that PRC2 as an epigenetic master regulator is involved in the regulation of numerous genes [21,39].

Interestingly, we also observed a non-significant increase in EZH2 protein expression in KKU-055 cells in some of the biological replicates following tazemetostat treatment. Since tazemetostat inhibits only the enzymatic activity and not the EZH2 expression, this observed elevation of the EZH2 expression in KKU-055 might be due to a compensatory reaction.

Based on the current literature, we therefore selected *n* = 21 EZH2 target genes and measured their mRNA levels after tazemetostat application. By doing this, we found in KKU-055 that mRNA and protein levels of FBP1, a key enzyme in the gluconeogenesis, were significantly upregulated. These findings are in accordance with the study carried out by Wang et al., which demonstrated that FBP1 is partly epigenetically silenced/regulated via EZH2 [40]. Furthermore, in silico analysis of the EZH2 and FBP1 mRNA expressions in human CCA samples revealed that the EZH2 mRNA expression was upregulated in tumor samples, whereas the FBP1 expression was downregulated. Interestingly previous studies already described a potential tumor suppressor role of FBP1 in BTC [40,41].

Wang et al. demonstrated that mRNA and the protein expression of FBP1 were lower in CCA tissue compared to adjacent non-tumor tissue [40].

Furthermore, in BTC cells, when the inhibition of FBP1 was abolished by si-EZH2, the proliferation and migration of CCA cells was depleted, whereas the forced overexpression of FBP1 inhibited proliferation, migration, metastasis as well as colony formation [40,41]. Additionally, Zhao et al. demonstrated that FBP1 might act as a possible tumor suppressor gene via the beta catenin way [41]. Thus, further studies are required to investigate the role of FBP1 in BTC cell lines.

A potential marker for the susceptibility of tumor cells towards tazemetostat could be the mutation status of EZH2. In previous studies, it was demonstrated that cells containing a point mutation, Y641/S or H within EZH2 are more sensitive to tazemetostat than the wild-type cells [22,33]. Up to now, these gain-of-function-mutations were mostly found in lymphomas. However, there are some descriptions of such mutations also in solid tumors. Tiffen et al., for example, found that mutated EZH2 (Y641) was constitutively active in melanoma [42]. Furthermore, this mutation was responsible for the silencing of tumor suppressor genes in melanoma [42].

In our study, we found Y641S mutations in OCUG-1 and TFK-1 cells. This is the first-time investigation of mutated EZH2 in BTC and in a solid tumor beside melanoma. However, based on the results of cell viability, we could not find any correlation between the mutated EZH2 and the susceptibility towards tazemetostat in OCUG-1 and TFK-1 cells. Given the fact that there are already EZH2 mutation kits available to test if patients are eligible for tazemetostat therapy in metastatic and/or locally advanced epithelioid sarcoma, it might be interesting for future studies to investigate the role of EZH2 mutation in BTC for potential diagnostic and therapeutic purposes [17,18,43]. In this regard, Morschhauser et al. confirmed an increased response rate in patients with relapsed or refractory follicular lymphoma harboring EZH2 mutations [44].

Cisplatin, a commonly used chemotherapeutic agent, is part of the standard therapeutic option for metastatic or locally advanced BTC [4]. However, BTC cells are often resistant to this intervention [4,13]. There might be evidence that tazemetostat can be used as an adjuvant therapeutic approach [20]. Furthermore, it was already demonstrated in several cancer entities that EZH2 might be involved in cisplatin resistance [45]. Therefore, EZH2 inhibition might sensitize resistant cells and/or enhance the cytotoxic effect of chemotherapeutics [46,47,48]. For example, Hu et al. could demonstrate that EZH2 was overexpressed in cisplatin-resistant ovarian cells compared to non-cisplatin-resistant cells [47]. Furthermore, EZH2 knockdown sensitized resistant ovarian cells towards cisplatin [47].

In another study, carried out by Cao et al., tazemetostat could enhance the cisplatin-induced apoptosis and cytotoxicity [46].

In our experimental setup, the treatment of BTC cells with tazemetostat did not augment the cytotoxicity of cisplatin, which might be explainable by tumor-specific phenomena. It would be interesting in future studies to investigate the effect of tazemetostat in combination with other commonly used chemotherapeutics such as 5-FU, doxorubicin and gemcitabine in BTC.

## 5. Conclusions

The current study represents the first approach to investigate the effect of tazemetostat on BTC cells. We found that tazemetostat impairs clonogenic growth, as well as cell viability following long-term incubation. Moreover, we found that tazemetostat has a strong epigenetic effect in BTC cells and significantly reduces H3K27me3 levels. Furthermore, we observed a cell line-specific up-regulation of the tumor suppressor gene FBP1 following tazemetostat application on mRNA and protein levels. Interestingly, we could also demonstrate that the EZH2 Y641 point mutations occur in BTC cells.

To conclude, our results provide the first evidence of tazemetostat as a possible anti-BTC agent and should be used as a base for further detailed investigations as well as in vivo experimentations.

## Figures and Tables

**Table 1:** Applied antibodies used in the immunohistochemical staining of three used BTC cell lines KKU-055, NOZ and OCUG-1.

Antibody	Vendor	Cat. -No.	Clone	Pre-Treatment	Dilution/ Incubation	Detection Kit	Platform
Cytokeratin 7	Ventana	598618001	Sp52	High pH	Ready-to-use (rtu)	Ultraview	Ventana
EZH2	Cell Signaling	5246S	D2C9	High pH	rtu	Ultraview	Ventana
Vimentin	Ventana	5278139001	V9	High pH	rtu	Ultraview	Ventana

**Table 2:** EZH2 mutation status of biliary tract cancer cell lines, where Y641S represents a gain-of-function mutation. n.d.: not defined.

BTC Cell Lines	Genotype	Mutational Status
EGI-1	TAC	wild type
HuCCT1	n.d.	n.d.
KKU-055	TAC	wild type
MMNK-1	TAC	wild type
NOZ	TAC	wild type
OCUG-1	TCC	Y641S
OZ	TAC	wild type
TFK-1	TCC	Y641S

## Data Availability

Available Biodata in this study are found on SMART App http://www.bioinfo-zs.com/smartapp (accessed on 26 January 2023), GEPIA http://gepia.cancer-pku.cn (accessed on 26 January 2023) and DNMIVD http://www.unimd.org/dnmivd/ (accessed on 26 January 2023). All other data of this study are available in this research article.

## Supplementary Materials

The following supporting information can be downloaded at: https://www.mdpi.com/article/10.3390/cancers15051569/s1, Figure S1: Primer table; Figure S2: EZH2 sequencing primer; Figure S3: clonogenic assay of remaining BTC cell lines; Figure S4: Tazemetostat and Cisplatin co-treatment; Figure S5: EZH2-associated genes table; Figure S6: In silico analysis of EZH2 and FBP1; Figure S7: Sanger Sequencing data of Y641 EZH2; Figure S8: Immunohistochemistry analysis; Figure S9: Original Images for Blots.
