# Supplementary material for: Evaluation of Tazemetostat as a Therapeutically Relevant Substance in Biliary Tract Cancer
Source: Cancers (Basel). 2023 Mar 2;15(5):1569. doi: 10.3390/cancers15051569 (PMC10000745; doi:10.3390/cancers15051569)
Supplement: Supplementary file 1 [file cancers-15-01569-s001.zip › cancers-2176140-supplementary.pdf]

Supplementary Figure S1 Primer Table of genes of interest

Batch #	Oligo Name	Oligo #	Len	Pur	Scale	MW	Tm°	μg/OD	OD	μg	nmol	Epsilon 1/(mMcm)	Dime	2ndry	GC %	μl for 100μM*	Sequence(5'-3')
ST04471164	FH1_KLF2	8814566191-70/0	20	RP1	0.025	6150	57.3	31.6	8.6	272.4	44.3	194.1	No	Very Weak	40	443	AAGGGCCTTAATTTGTACTG
																	Forward Human 1 KLF2
ST04471165	RH1_KLF2	8814566191-70/1	20	RP1	0.025	6156	57.3	28.9	8.5	245.6	39.9	213	No	None	40	399	ATAAAAACGAACCAGGTAGC
																	Reverse Human 1 KLF2
ST04471166	FH1_ABI3BP	8814566191-80/0	22	RP1	0.025	6722	57.0	31.3	9.7	304.2	45.2	214.3	No	Weak	40.9	452	CCAGGTCTTAATGAAACTACTG
																	Forward Human 1 ABI3BP
ST04471167	RH1_ABI3BP	8814566191-80/1	20	RP1	0.025	6181	54.4	31.7	8.3	263.7	42.6	194.5	No	None	40	426	AGCTAATTGTGTCTTGAGTG
																	Reverse Human 1 ABI3BP
ST04471168	FH1_LATS2	8814566191-90/0	20	RP1	0.025	6119	57.4	31.3	8.7	272.5	44.5	195.3	No	Very Weak	40	445	TGGATGGTCACATTAAACTC
																	Forward Human 1 LATS2
ST04471169	RH1_LATS2	8814566191-90/1	22	RP1	0.025	6685	60.3	33.7	9.8	330.7	49.4	198.1	No	None	40.9	494	CTCCCTTTCTGGTAATATTTGG
																	Reverse Human 1 LATS2
ST04471170	FH1_FBP1	8814566191-100/0	22	RP1	0.025	6673	59.3	31.9	9.7	309.5	46.3	209.1	No	Very Weak	40.9	463	CCAGATAATTCAGCTCCTTATG
																	Forward Human 1 FBP1
ST04471171	RH1_FBP1	8814566191-100/1	20	RP1	0.025	6073	56.2	30.4	9.1	277.0	45.6	199.5	No	None	45	456	AGAAATATCCCTCCGTAGAC
																	Reverse Human 1 FBP1
ST04471172	FH1_MUC1	8814566191-110/0	22	RP1	0.025	6673	58.4	31.8	8.8	279.8	41.9	209.8	No	None	40.9	419	GCCTCTCCAATATTAAGTTCAG
																	Forward Human 1 MUC1
ST04471173	RH1_MUC1	8814566191-110/1	21	RP1	0.025	6489	57.3	30.0	9.5	285.5	44.0	215.9	No	Weak	42.8	440	AGATCGTCAGGTTATATCGAG
																	Reverse Human 1 MUC1
ST04471174	FH1_MUC6	8814566191-120/0	20	RP1	0.025	6061	58.1	33.0	9.3	307.3	50.7	183.4	No	Very Weak	40	507	CTTTGTTACCACATTTGACG
																	Forward Human 1 MUC6
ST04471175	RH1_MUC6	8814566191-120/1	20	RP1	0.025	6070	59.2	32.2	9.2	296.4	48.8	188.4	No	Very Weak	40	488	AGATCACAATTTTGTCCTGC
																	Reverse Human 1 MUC6

Batch #	Oligo Name	Oligo #	Len	Pur	Scale	MW	Tm°	μg/OD	OD	μg	nmol	Epsilon 1/(mMcm)	Dime	2ndry	GC %	μl for 100μM*	Sequence(5'-3')
ST04681976	FH1_PGK1	8815306723-70/0	20	RP1	0.025	6154	60.6	30.7	9.3	285.5	46.4	200.4	No	Weak	45	464	CTCAACAACATGGAGATTGG
																	Forward Human 1 PGK1
ST04681977	RH1_PGK1	8815306723-70/1	22	RP1	0.025	6734	59.2	32.8	10.6	348.5	51.7	204.8	No	Weak	40.9	517	CTTTGGACATTAGGTCTTTGAC
																	Reverse Human 1 PGK1
ST04681978	FH1_SLC2A1	8815306723-80/0	20	RP1	0.025	6110	61.2	31.9	11.2	357.9	58.5	191.2	No	None	40	585	ACCTCAAATTTCATTGTGGG
																	Forward Human 1 SLC2A1
ST04681979	RH1_SLC2A1	8815306723-80/1	21	RP1	0.025	6534	57.9	28.3	9.6	272.1	41.6	230.5	No	None	42.8	416	GAAGATGAAGAACAGAACCAG
																	Reverse Human 1 SLC2A1
ST04681980	FH1_EAF2	8815306723-90/0	21	RP1	0.025	6319	58.1	33.4	9.5	317.8	50.2	188.9	No	None	42.8	502	CTATGACTTCAAACCTGCTTC
																	Forward Human 1 EAF2
ST04681981	RH1_EAF2	8815306723-90/1	20	RP1	0.025	6126	55.5	31.8	10.7	340.6	55.6	192.4	No	None	45	556	AGAGTTATGGTCACCTGTTC
																	Reverse Human 1 EAF2
ST04681982	FH1_E2F1	8815306723-100/0	22	RP1	0.025	6713	55.6	31.7	9.6	304.4	45.3	211.7	No	Very Weak	40.9	453	CTGATGAATATCTGTACTACGC
																	Forward Human 1 E2F1
ST04681983	RH1_E2F1	8815306723-100/1	22	RP1	0.025	6633	60.8	32.3	8.4	272.0	41.0	204.8	No	Very Weak	40.9	410	CTTTGATCACCATAACCATCTG
																	Reverse Human 1 E2F1
ST04681984	FH1_JAK2	8815306723-110/0	18	RP1	0.025	5487	61.5	31.6	8.0	253.2	46.1	173.3	No	None	50	461	CCACCAGCGGAATTTATG
																	Forward Human 1 JAK2
ST04681985	RH1_JAK2	8815306723-110/1	20	RP1	0.025	6052	57.7	33.4	11.1	371.3	61.3	180.9	No	Weak	40	613	TATTGTTCCAGCATTCTGTC
																	Reverse Human 1 JAK2
ST04681986	FH1_VGLL4	8815306723-120/0	19	RP1	0.025	5788	55.4	29.2	10.5	306.9	53.0	198	No	None	47.3	530	AGAACCCAGAATACAGACC
																	Forward Human 1 VGLL4
ST04681987	RH1_VGLL4	8815306723-120/1	18	RP1	0.025	5589	57.5	31.6	9.4	297.8	53.2	176.4	No	Very Weak	50	532	AGTCTTGTTCAGATGGGG
																	Reverse Human 1 VGLL4

Batch #	Oligo Name	Oligo #	Len	Pur	Scale	MW	Tm°	μg/OD	OD	μg	nmol	Epsilon 1/(mMcm)	Dime	2ndry	GC %	μl for 100μM*	Sequence(5'-3')
ST04681964	FH1_SLC31A1	8815306723-10/0	20	RP1	0.025	6046	56.7	33.1	8.6	285.2	47.1	182.3	No	None	45	471	TGATGCCTATGACCTTCTAC
																	Forward Human 1 SLC31A1
ST04681965	RH1_SLC31A1	8815306723-10/1	21	RP1	0.025	6439	56.4	32.2	9.3	300.1	46.6	199.5	No	None	42.8	466	GAATGCTGACTTGTGACTTAC
																	Reverse Human 1 SLC31A1
ST04681966	FH1_FOXO1	8815306723-20/0	20	RP1	0.025	6132	55.5	29.3	10.1	296.0	48.2	209.2	No	Weak	45	482	GTCAAGACAACGACACATAG
																	Forward Human 1 FOXO1
ST04681967	RH1_FOXO1	8815306723-20/1	20	RP1	0.025	6258	57.3	29.4	8.7	256.0	40.9	212.6	No	Weak	40	409	AAACTAAAAGGGAGTTGGTG
																	Reverse Human 1 FOXO1
ST04681968	FH1_G6PC	8815306723-30/0	20	RP1	0.025	6070	55.8	31.9	6.1	194.6	32.0	190.2	No	Very Weak	40	320	ACTGTGCATACATGTTCATC
																	Forward Human 1 G6PC
ST04681969	RH1_G6PC	8815306723-30/1	20	RP1	0.025	6141	57.8	32.4	8.8	285.6	46.5	189.2	No	None	40	465	TGAATGTTTTGACCTAGTGC
																	Reverse Human 1 G6PC
ST04681970	FH1_HK2	8815306723-40/0	21	RP1	0.025	6418	54.0	30.8	9.6	296.3	46.1	207.9	No	Weak	42.8	461	CTAAACTAGACGAGAGTTTCC
																	Forward Human 1 HK2
ST04681971	RH1_HK2	8815306723-40/1	20	RP1	0.025	6033	57.7	30.9	9.5	294.2	48.7	194.8	No	Weak	45	487	CATCATAACCACAGGTCATC
																	Reverse Human 1 HK2
ST04681972	FH1_LDHA	8815306723-50/0	22	RP1	0.025	6713	58.9	31.6	10.4	329.4	49.0	211.9	No	Weak	40.9	490	CACCATGATTAAGGGTCTTTAC
																	Forward Human 1 LDHA
ST04681973	RH1_LDHA	8815306723-50/1	20	RP1	0.025	6126	58.2	32.0	10.1	323.4	52.7	191.3	No	Very Weak	45	527	AGGTCTGAGATTCCATTCTG
																	Reverse Human 1 LDHA
ST04681974	FH1_PDK1	8815306723-60/0	20	RP1	0.025	6079	60.7	31.2	11.2	349.5	57.4	194.8	No	Weak	40	574	ATGATGTCATTCCCACAATG
																	Forward Human 1 PDK1
ST04681975	RH1_PDK1	8815306723-60/1	20	RP1	0.025	6209	53.4	29.9	10.1	302.7	48.7	207.1	No	None	40	487	AAGAGTGCTGATTGAGTAAC
																	Reverse Human 1 PDK1

Batch #	Oligo Name	Oligo #	Len	Pur	Scale	MW	Tm°	μg/OD	OD	μg	nmol	Epsilon 1/(mMcm)	Dime	2ndry	GC %	μl for 100μM*	Sequence(5'-3')
ST04471152	FH1_RUNX3	8814566191-10/0	18	RP1	0.025	5496	55.0	30.6	8.4	257.3	46.8	179.4	No	Weak	50	468	ATGACGAGAACTACTCCG
																	Forward Human 1 RUNX3
ST04471153	RH1_RUNX3	8814566191-10/1	18	RP1	0.025	5478	57.6	32.6	7.3	238.0	43.4	168	No	Very Weak	50	434	TCAGGGTGAAACTCTTCC
																	Reverse Human 1 RUNX3
ST04471154	FH1_CDH1	8814566191-20/0	17	RP1	0.025	5158	56.8	32.1	7.6	244.2	47.3	160.5	No	None	58.8	473	CCGAGAGCTACACGTTC
																	Forward Human 1 CDH1
ST04471155	RH1_CDH1	8814566191-20/1	20	RP1	0.025	5990	59.4	33.5	8.4	281.5	47.0	178.7	No	None	40	470	TCTTCAAAATTCACTCTGCC
																	Reverse Human 1 CDH1
ST04471156	FH1_CDKN1A	8814566191-30/0	20	RP1	0.025	6064	57.3	31.6	8.4	265.8	43.8	191.6	No	None	45	438	CAGCATGACAGATTTCTACC
																	Forward Human 1 CDKN1A
ST04471157	RH1_CDKN1A	8814566191-30/1	20	RP1	0.025	6249	57.0	29.8	7.5	224.1	35.8	209.1	No	None	50	358	CAGGGTATGTACATGAGGAG
																	Reverse Human 1 CDKN1A
ST04471158	FH1_CDKN1B	8814566191-40/0	20	RP1	0.025	6015	57.4	32.5	6.5	211.5	35.1	184.8	No	None	45	351	AACCGACGATTCTTCTACTC
																	Forward Human 1 CDKN1B
ST04471159	RH1_CDKN1B	8814566191-40/1	20	RP1	0.025	6083	57.8	33.9	8.8	298.5	49.0	179.3	No	Moderate	40	490	TGTTTACGTTTGACGTCTTC
																	Reverse Human 1 CDKN1B
ST04471160	FH1_CDKN2A	8814566191-50/0	15	RP1	0.025	4596	58.4	32.0	7.0	224.4	48.8	143.3	No	None	60	488	AGCATGGAGCCTTCG
																	Forward Human 1 CDKN2A
ST04471161	RH1_CDKN2A	8814566191-50/1	17	RP1	0.025	5213	59.4	31.4	8.1	254.3	48.7	166	No	Very Weak	52.9	487	ATCATGACCTGGATCGG
																	Reverse Human 1 CDKN2A
ST04471162	FH1_PTEN	8814566191-60/0	21	RP1	0.025	6507	57.8	29.9	9.0	269.2	41.3	217.5	No	Very Weak	42.8	413	GGCTAAGTGAAGATGACAATC
																	Forward Human 1 PTEN
ST04471163	RH1_PTEN	8814566191-60/1	21	RP1	0.025	6323	57.0	35.4	8.8	312.2	49.3	178.2	No	None	42.8	493	GTTACTCCCTTTTTGTCTCTG
																	Reverse Human 1 PTEN

Batch #	Oligo Name	Oligo #	Len	Pur	Scale	MW	Tm°	μg/OD	OD	μg	nmol	Epsilon 1/(mMcm)	Dime	2ndry	GC %	μl for 100μM*	Sequence(5'-3')
ST04681988	FH1_ACE2	8815306723-130/0	22	RP1	0.025	6682	58.8	31.1	9.4	292.4	43.7	214.8	No	Very Weak	40.9	437	GTTTGTAACCCAGATAATCCAC
																	Forward Human 1 ACE2
ST04681989	RH1_ACE2	8815306723-130/1	20	RP1	0.025	6061	61.6	33.2	11.0	365.7	60.3	182.3	No	Very Weak	40	603	AATGATTTGCTCTTGCCATC
																	Reverse Human 1 ACE2
ST04681990	FH1_PCK1	8815306723-140/0	20	RP1	0.025	6064	59.1	31.4	13.0	408.4	67.3	193	No	Strong	45	673	ATTCTGGGTATAACCAACCC
																	Forward Human 1 PCK1
ST04681991	RH1_PCK1	8815306723-140/1	20	RP1	0.025	6135	60.8	31.9	9.8	312.8	50.9	192.2	No	None	45	509	GTTGATGGCCCTTAAATGAC
																	Reverse Human 1 PCK1

Supplementary Figure S2 EZH2 Primer for Sanger Sequencing

		Sequence (5'->3')	Template strand	Length	Start	Stop	Tm	GC%	[illegible] complement arity	[illegible] complement arity
Primer pair 5	Forward	TCGTCGGCAGCGTCAGATGTGTATAAGAGACAGTCCCCAGTCCATTTTCACCCT	Plus	21	77573	77593	61,1	52,38	2	0
	Reverse primer	GTCTCGTGGGCTCGGAGATGTGTATAAGAGACAGCCAATCAAACCCACAGACTTACCT	Minus	24	77822	77799	60,8	45,83	3	0
	Product length	250 + Adaptersequenzen								

Adaptersequences

Genspecific Primer

Supplementary Figure S3

Clonogenic Assay following tazemetostat treatment for 7 days in BTC cell lines

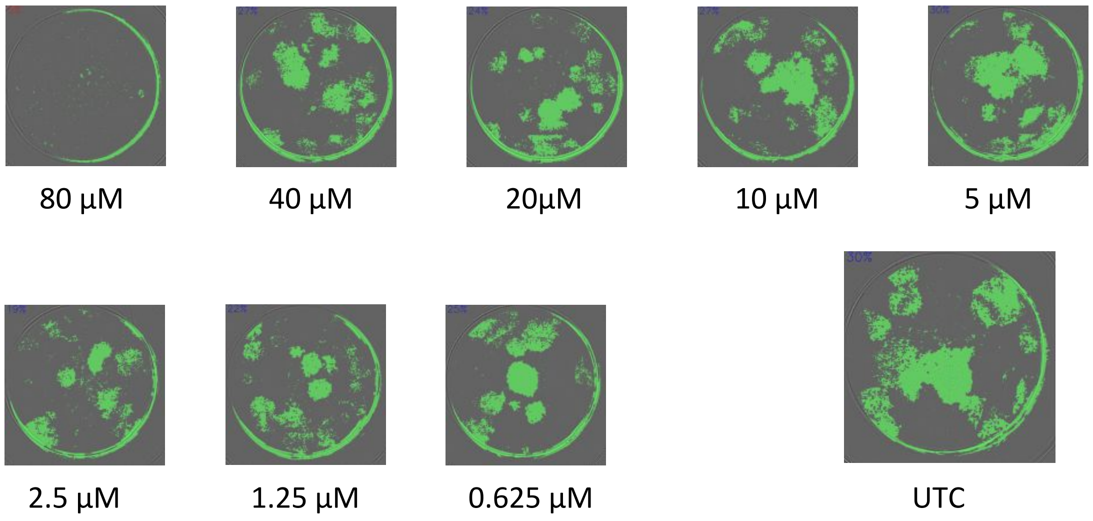

S3A Clonogenic Assay MMNK-1 after tazemetostat treatment for 7 days

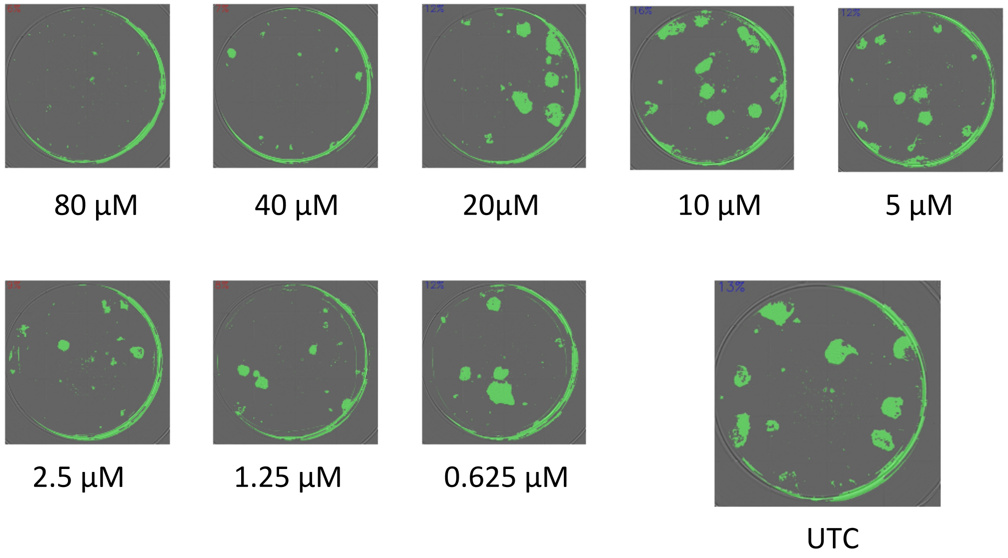

S3C Clonogenic Assay HuCCT1 after tazemetostat treatment for 7 days

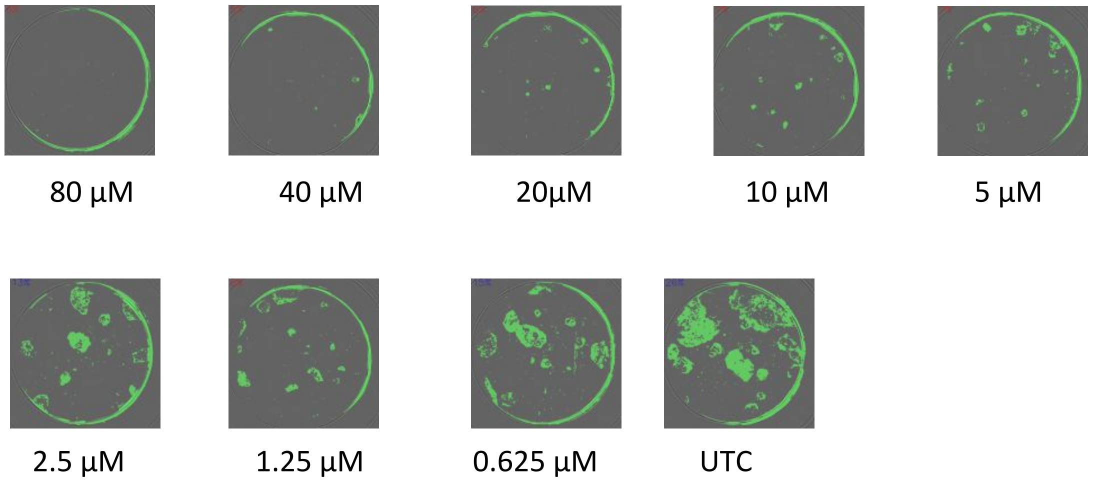

S3B Clonogenic Assay EGI-1 after tazemetostat treatment for 7 days

Supplementary Figure S4 Cell viability after tazemetostat plus cisplatin treatment

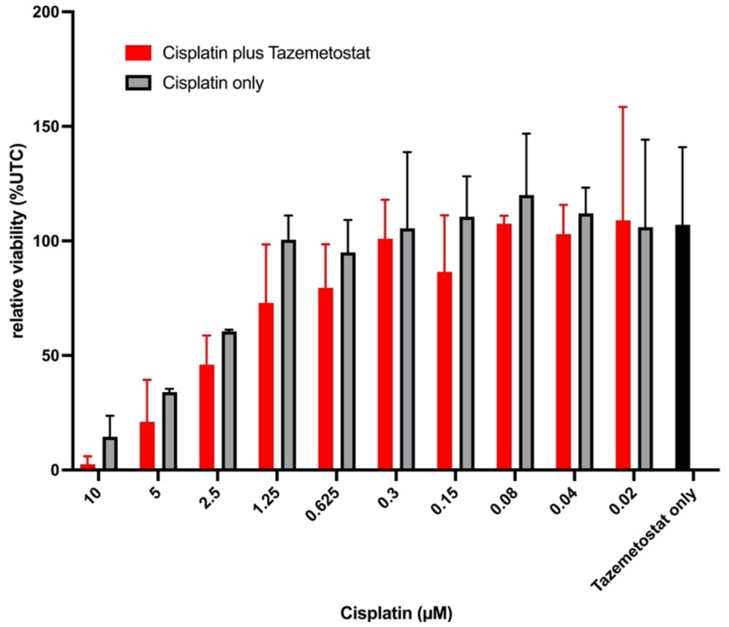

S4A KKU-055 cell viability compared to UTC after cisplatin + tazemetostat (30 µM) simultaneous treatment for 72 h. n= 3; SEM

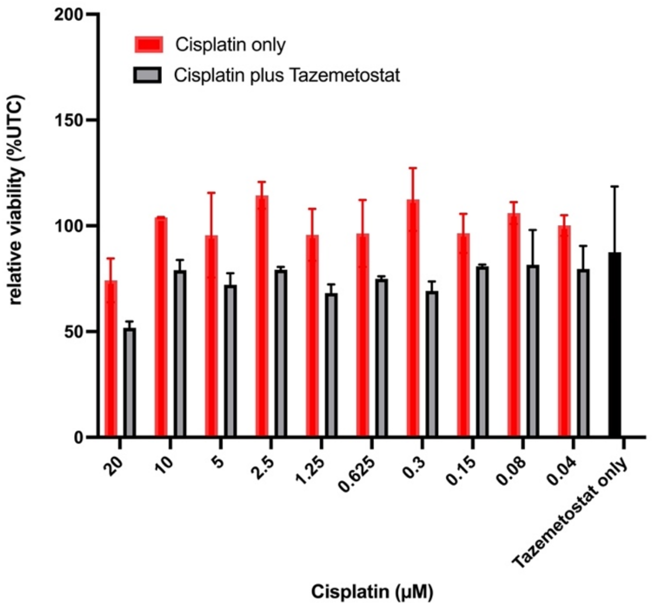

S4B NOZ cell viability compared to UTC after cisplatin + tazemetostat (30 µM) simultaneous treatment for 72 h. n= 3; SEM

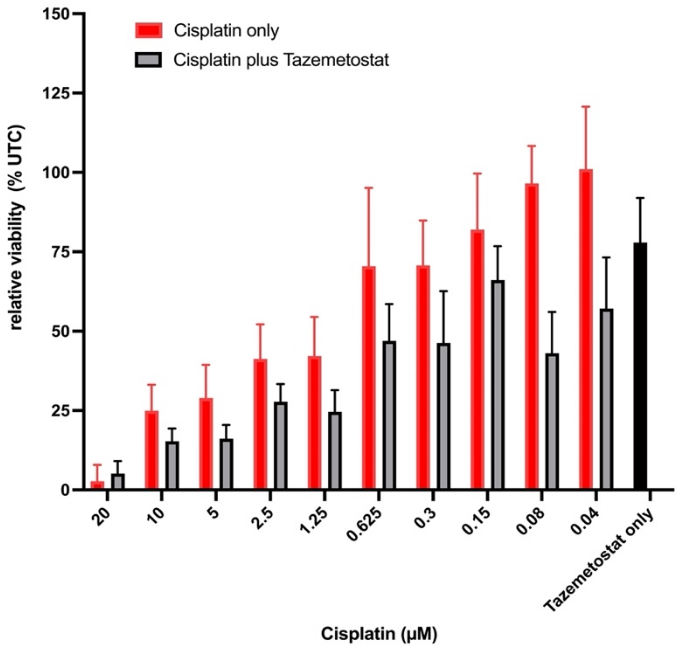

S4C KKU-055 cell viability compared to UTC after tazemetostat (30 µM) pre-treatment for 120 h followed by 72 h cisplatin only. n= 3; SEM

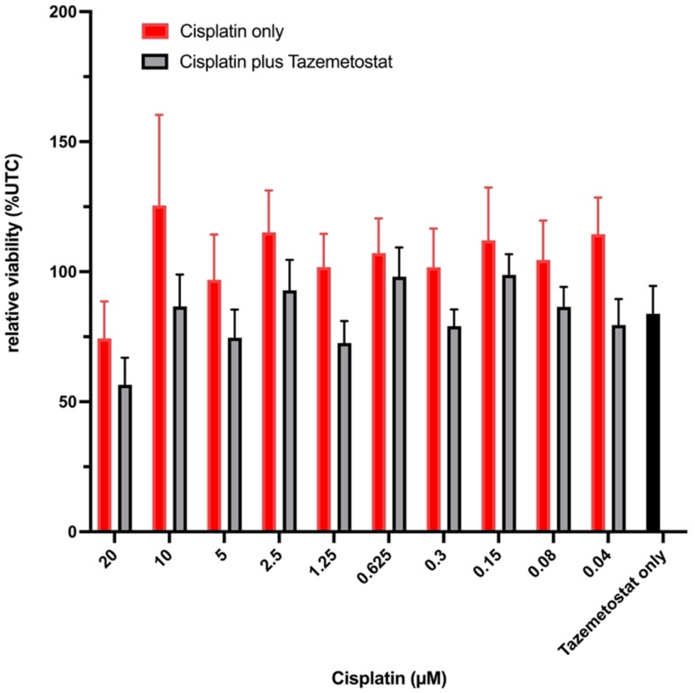

S4D NOZ cell viability compared to UTC after tazemetostat (30 µM) pre-treatment for 120 h followed by 72 h cisplatin only. n= 3; SEM

Supplementary Figure S5
EZH2 associated Genes

Gene of interest	Role in Cancer	Reference
ABI3BP	suppressor	Long noncoding RNA MALAT1 potentiates growth and inhibits senescence by antagonizing ABI3BP in gallbladder cancer cells
CDKN1A	suppressor	Epigenetic silencing of tumor suppressor gene CDKN1A by oncogenic long non-coding RNA SNHG1 in cholangiocarcinoma
CDKN1B	suppressor	Enhancer of zeste homolog 2 (EZH2) promotes progression of cholangiocarcinoma cells by regulating cell cycle and apoptosis.
CDKN2A	suppressor	Enhancer of zeste homolog 2 (EZH2) promotes progression of cholangiocarcinoma cells by regulating cell cycle and apoptosis.
E2F1	oncogene	EZH2 cooperates with E2F1 to stimulate expression of genes involved in adrenocortical carcinoma aggressiveness
EAF2	oncogene	EZH2 promotes metabolic reprogramming in glioblastomas through epigenetic repression of EAF2-HIF1α signaling
FBP1	suppressor	Long noncoding RNA DANCR regulates proliferation and migration by epigenetically silencing FBP1 in tumorigenesis of cholangiocarcinoma.
FOXO1	suppressor	EZH2 promotes invasion and tumour glycolysis by regulating STAT3 and FoxO1 signalling in human OSCC cells
HK2	oncogene	Involvement of EZH2 in aerobic glycolysis of prostate cancer through miR-181b/HK2 axis
JAK2	oncogene	EZH2 inhibition suppresses bladder cancer cell growth and metastasis via the JAK2/STAT3 signalling pathway
KLF2	suppressor	LINC00702 accelerates the progression of ovarian cancer through interacting with EZH2 to inhibit the transcription of KLF2
LATS2	suppressor	Long noncoding RNA MEG3 regulates LATS2 by promoting the ubiquitination of EZH2 and inhibits proliferation and invasion in gallbladder cancer
LDHA		Key enzymes of glycolysis
MUC1	oncogene	Overexpression of enhancer of zeste homolog 2 and MUC1 may be related to malignant behaviour in intraductal papillary neoplasm of the bile duct
PDK1	?	Repression of PDK1- and LncRNA HOTAIR-Mediated EZH2 Gene Expression Contributes to the Enhancement of Atractylenolide 1 and Erlotinib in the Inhibition of Human Lung Cancer Cells
PGK1	?	Key enzymes of glycolysis
PTEN	suppressor	FOXF2 aggravates the progression of non-small cell lung cancer through targeting lncRNA H19 to downregulate PTEN.
RUNX3	suppressor	SP1-induced upregulation of lncRNA SPRY4-IT1 exerts oncogenic properties by scaffolding EZH2/LSD1/DNMT1 and sponging miR-101-3p in cholangiocarcinoma
SLC2A1	?	Glucose Uptake
SLC31A0	?	Copper Transporter Cisplatin?<br><br>Enhancer of zeste homolog 2 promotes cisplatin resistance by reducing cellular platinum accumulation
VGLL4	?	Genome-wide expression analysis reveals six contravened targets of EZH2 associated with breast cancer patient survival

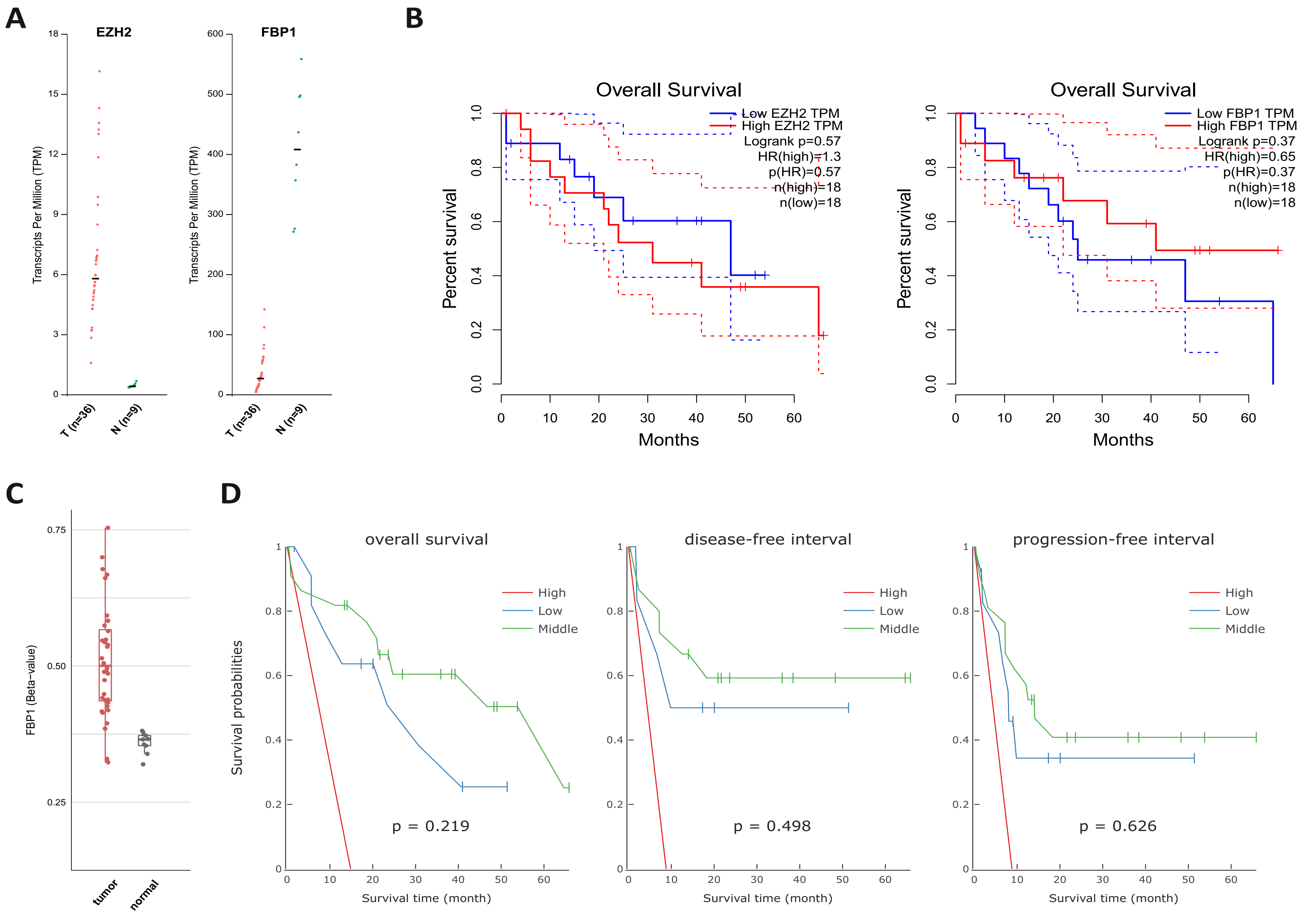

Supplementary Figure S6 In silico analysis data of EZH2 and FBP1 mRNA expression and DNA methylation of FBP1 in human BTC samples and clinical outcome
(A) mRNA expression of EZH2 and FBP1 in BTC samples versus normal samples **(B)** Kaplan Meyer Survival Curve comparison between high and low TPM of EZH2 as well as high and low TPM of FBP1 in BTC samples. EZH2: p = 0.57; FPB1: p= 0.37 **(C)** DNA methylation status of FBP1 in BTC samples versus normal samples
(**D**) Kaplan Meyer Survival Curve, disease-free interval, and progression-free interval comparison between high, middle and low DNA methylation status of FBP1. Overall Survival: p= 0.219, Disease-free interval: p= 0.498, Progression-free interval: p= 0.626

Supplementary Figure S7 Sanger Sequencing of EZH2 Y641

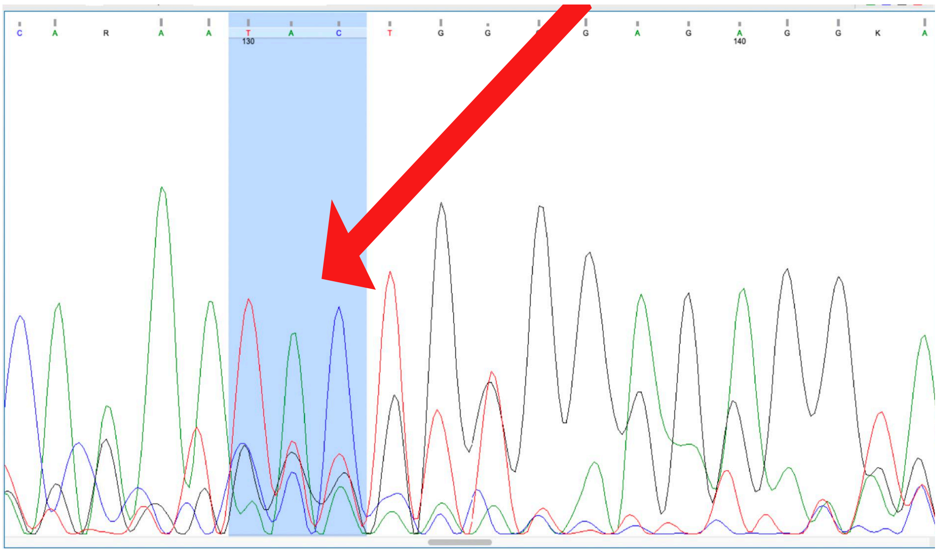

S7A NOZ TAC Wildtype (Y641)

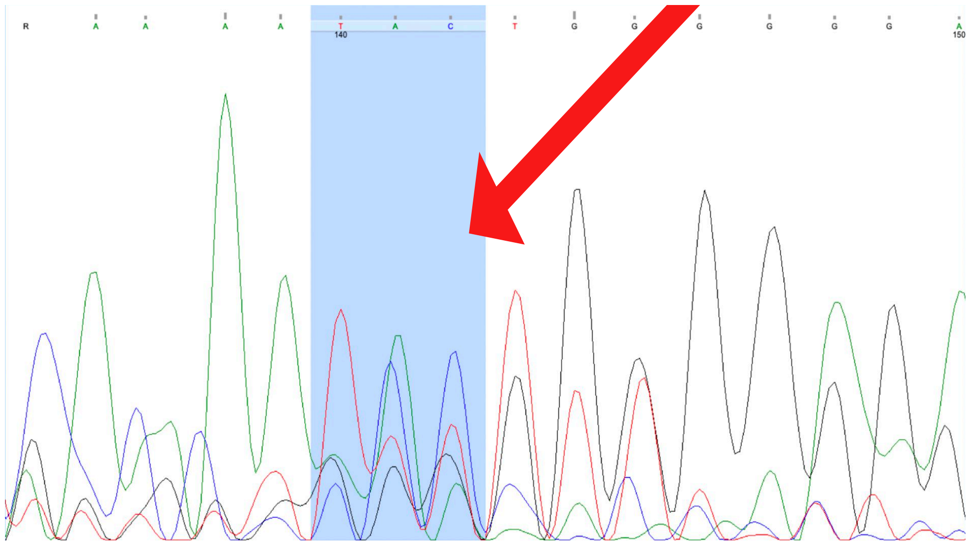

S7B OCUG-1 TCC Mutant (Y641S)

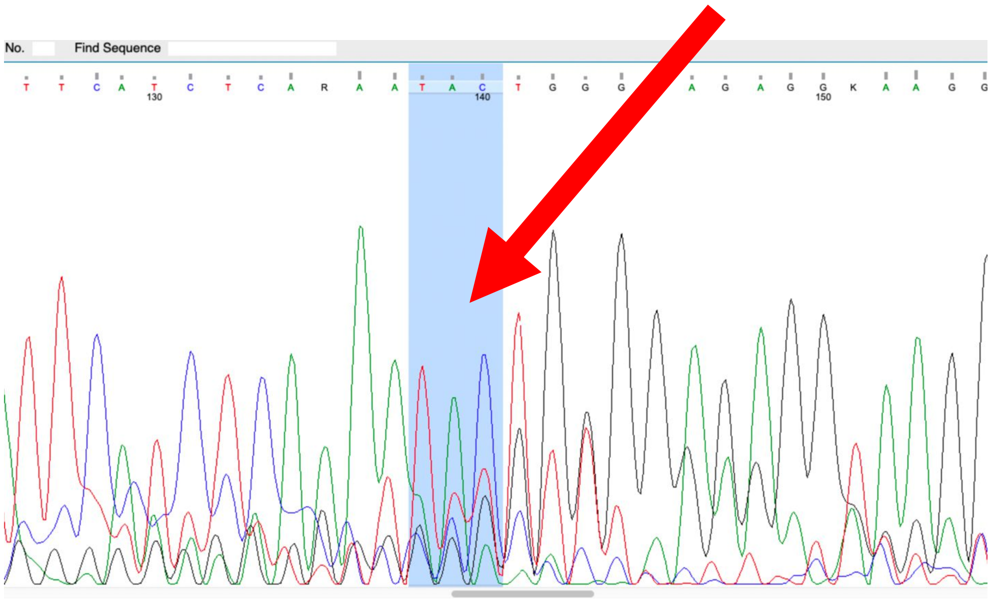

S7C EGI-1 TAC Wildtype (Y641)

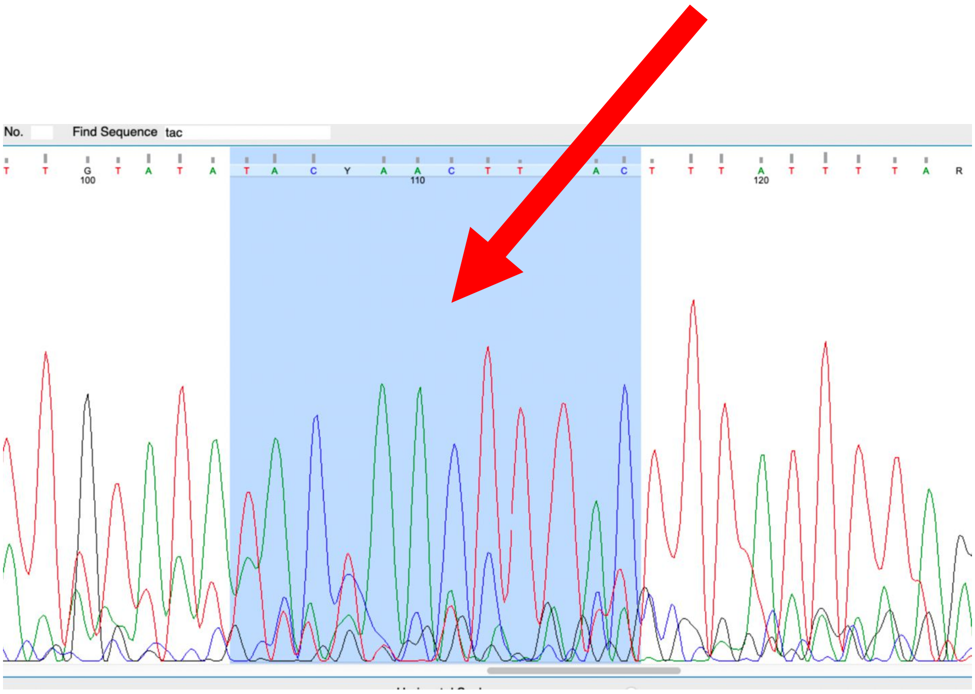

S7D HuCCT-1 n.d.

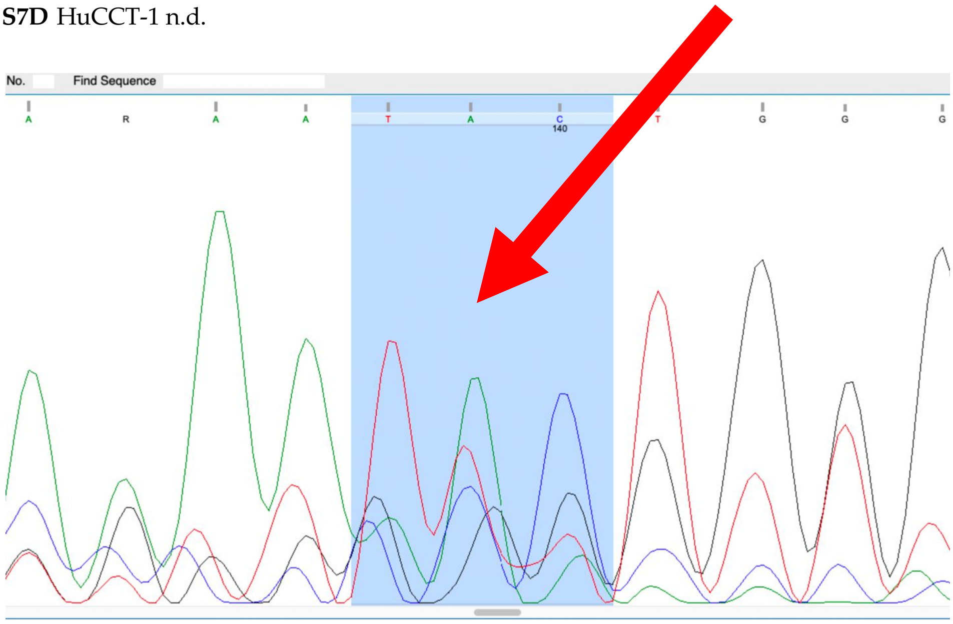

S7E KKU-055 TAC Wildtype (Y641)

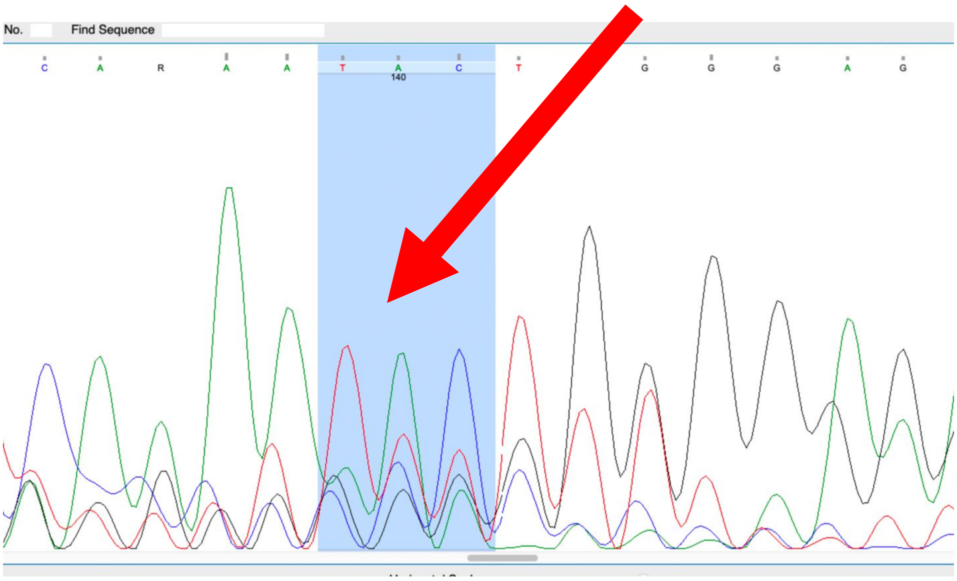

S7F MMNK-1 TAC Wildtype (Y641)

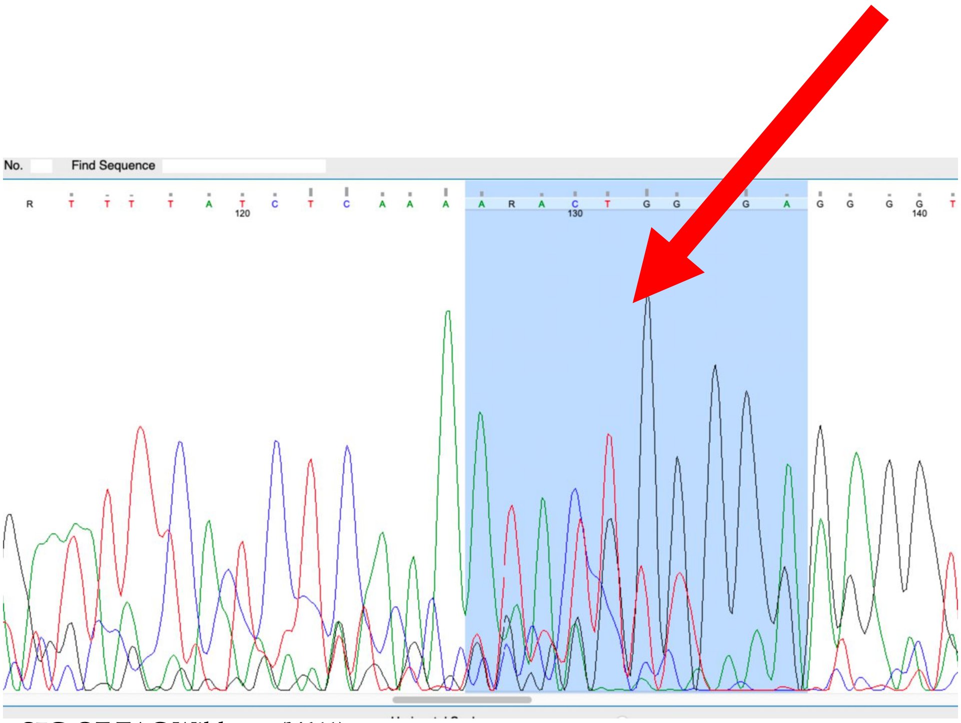

S7G OZ TAC Wildtype (Y641)

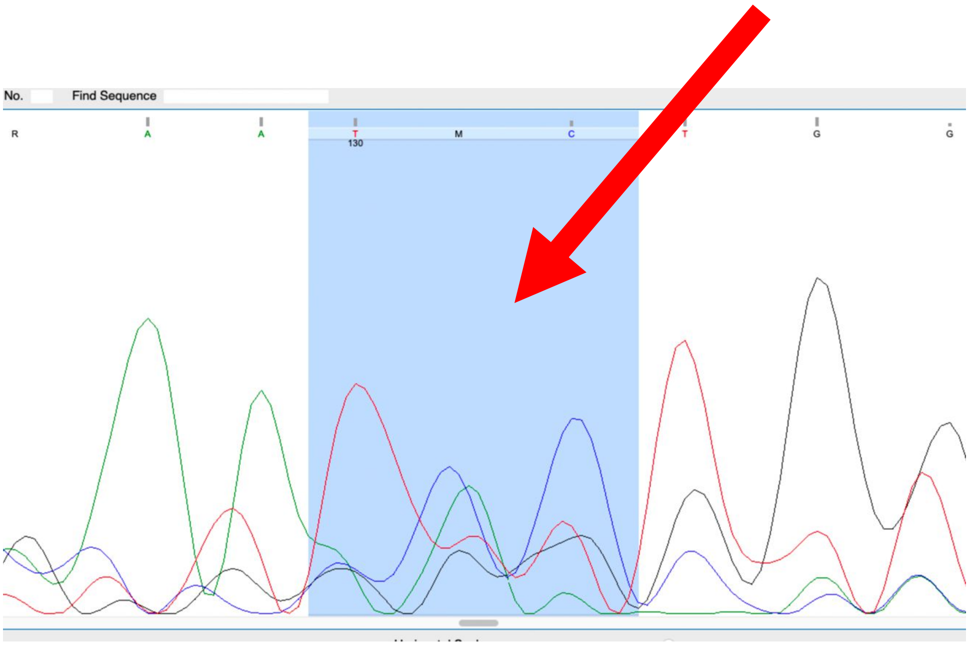

S7H TFK-1 TCC Mutant (Y641S)

Supplementary Figure S8 Determination of baseline EZH2 expression and Epithelial & Mesenchymal phenotype via Immunohistochemistry

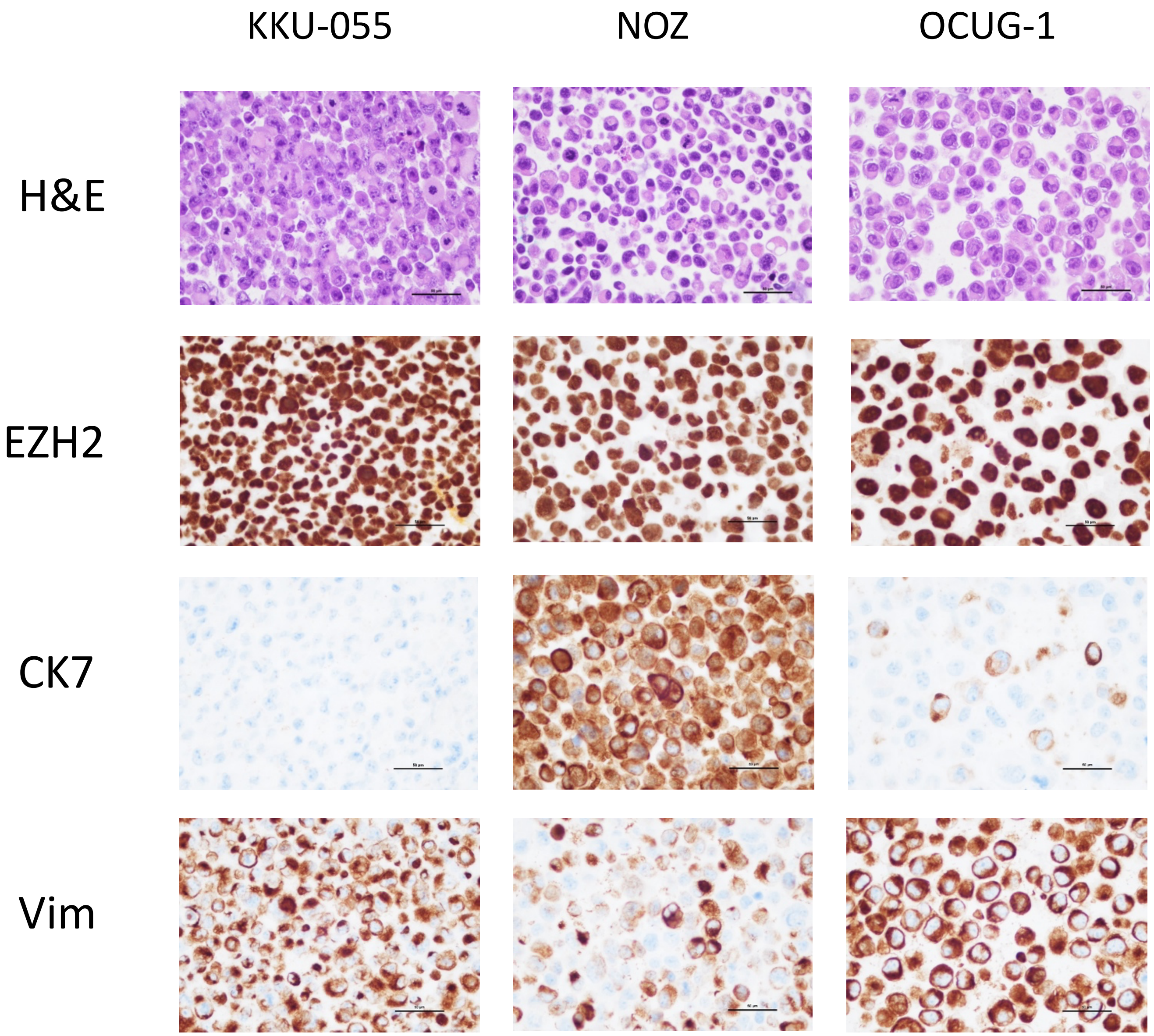

H&E = Hematoxylin and Eosin; CK7 = Cytokeratin 7; EZH2 = Enhancer of Zeste Homolog 2; Vim = Vimentin
