# Supplementary figures and images for: Evaluation of Tazemetostat as a Therapeutically Relevant Substance in Biliary Tract Cancer
Source: Cancers (Basel). 2023 Mar 2;15(5):1569. doi: 10.3390/cancers15051569 (PMC10000745; doi:10.3390/cancers15051569)

Figure 2C

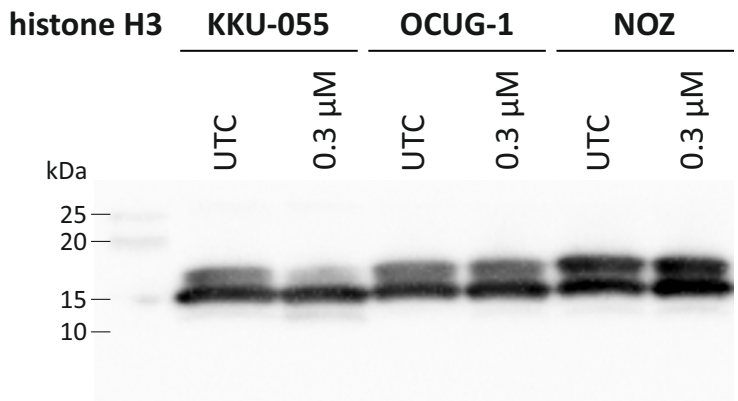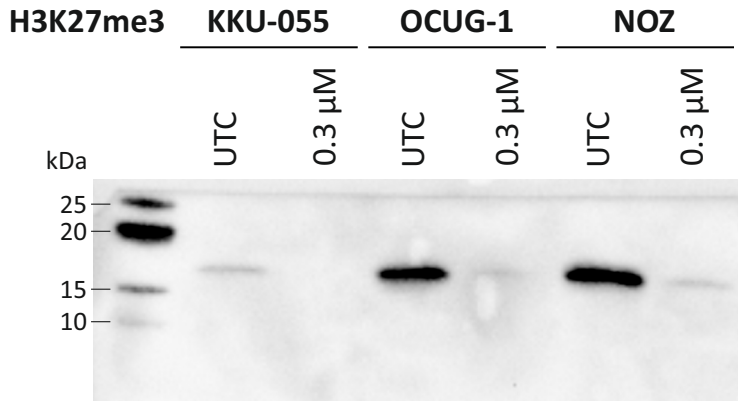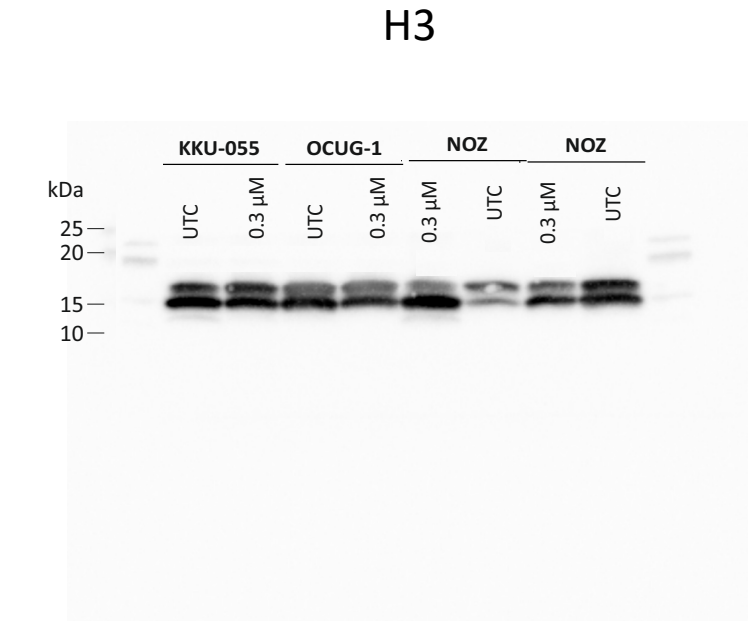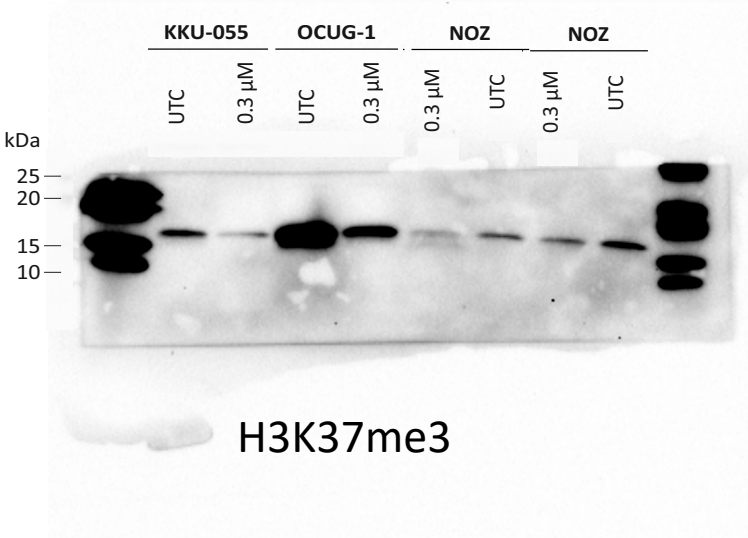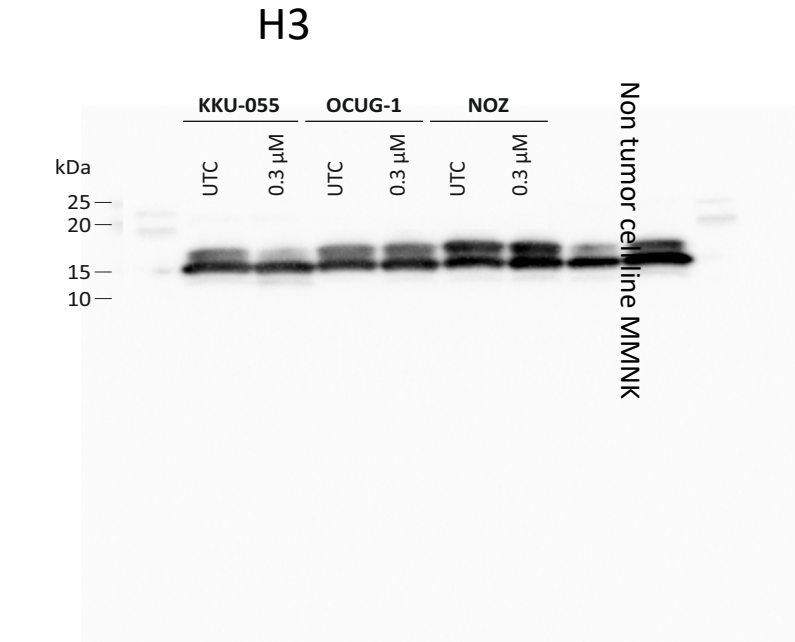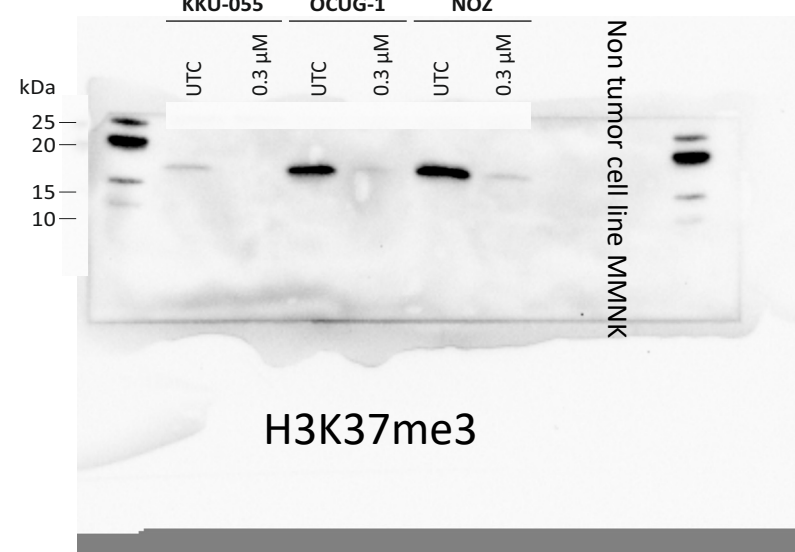

# Figure 2C

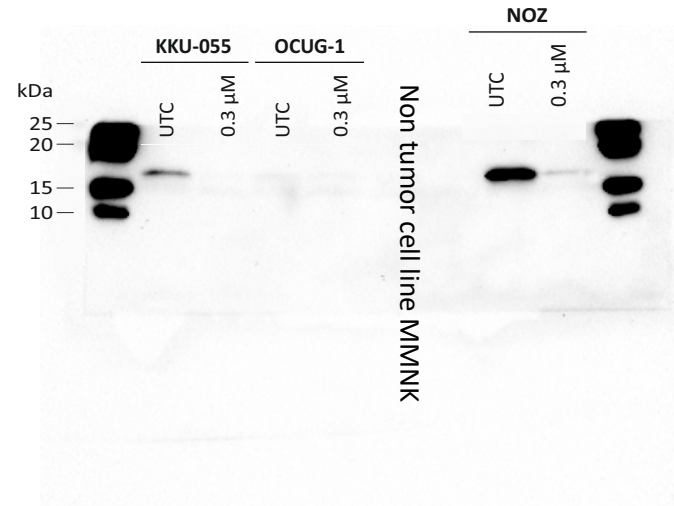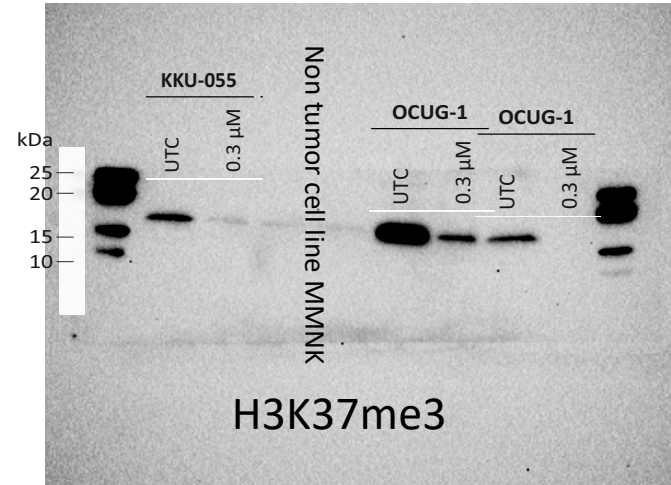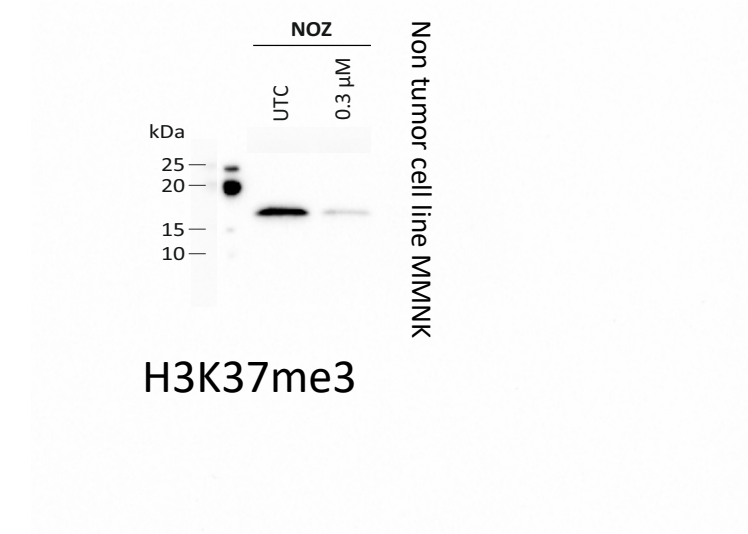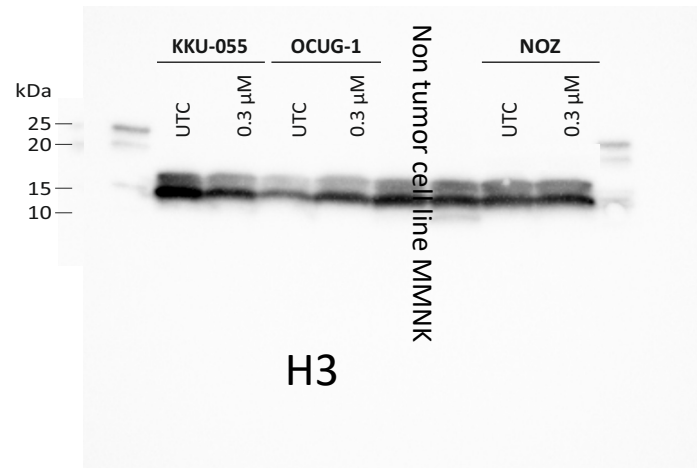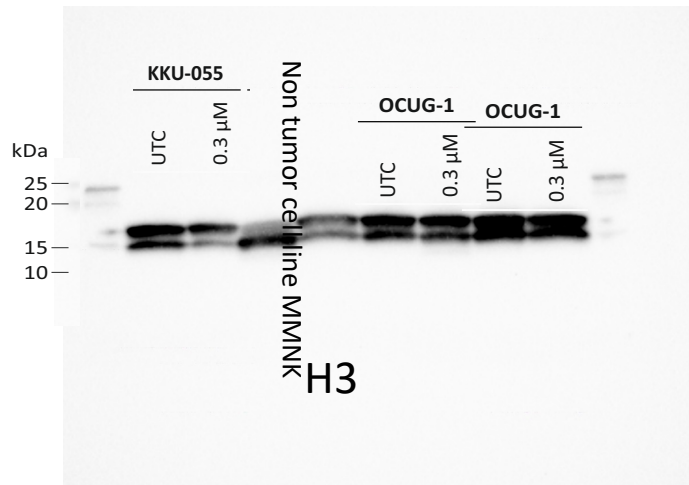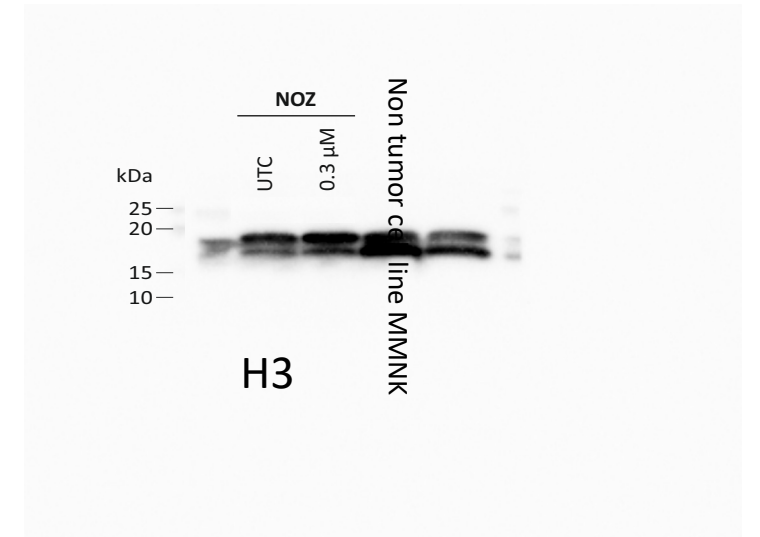

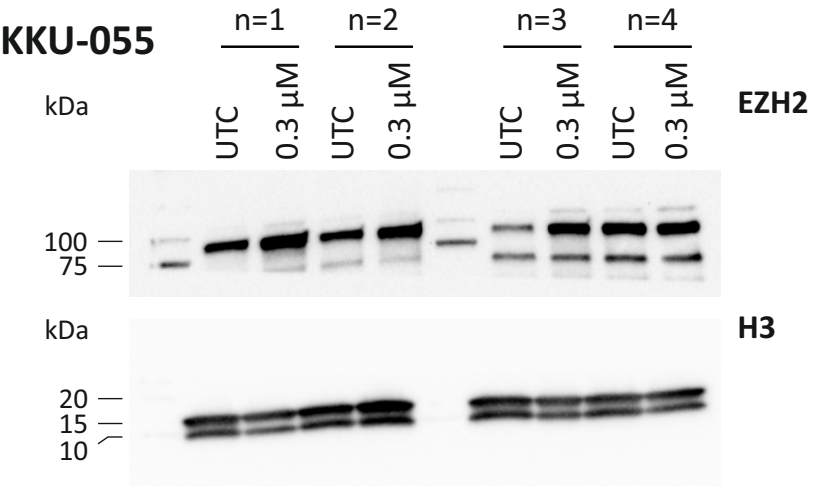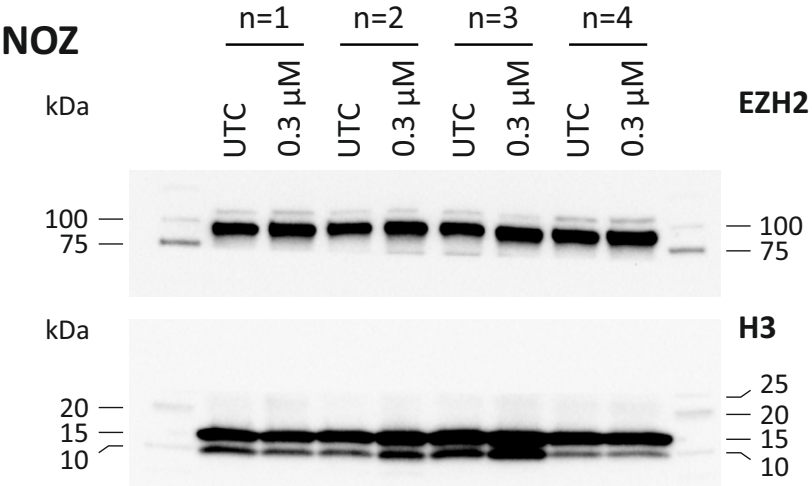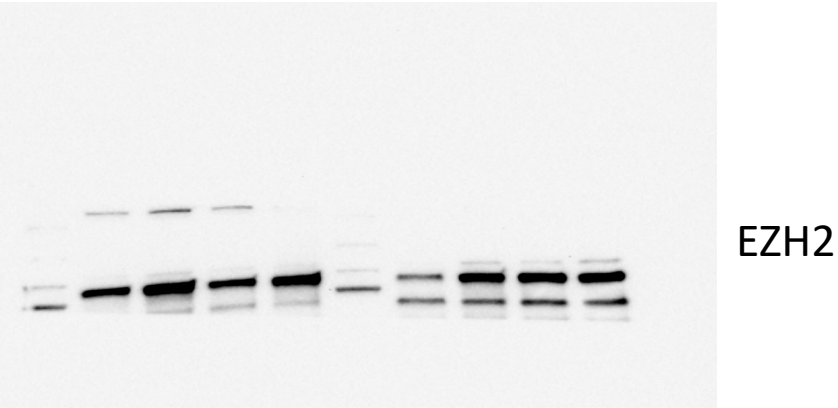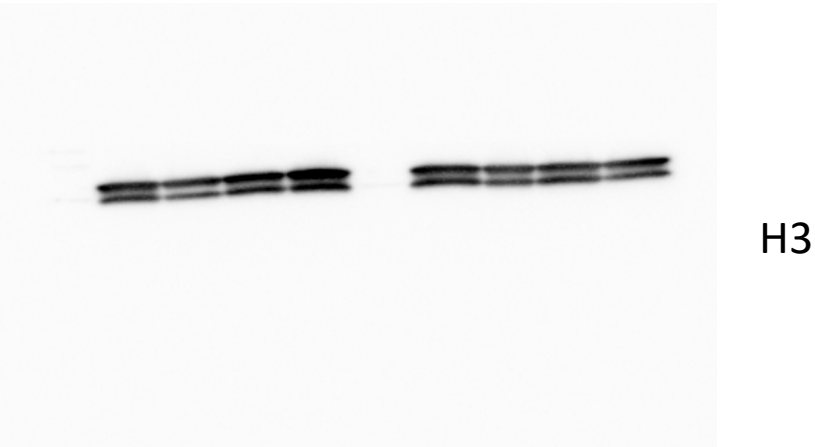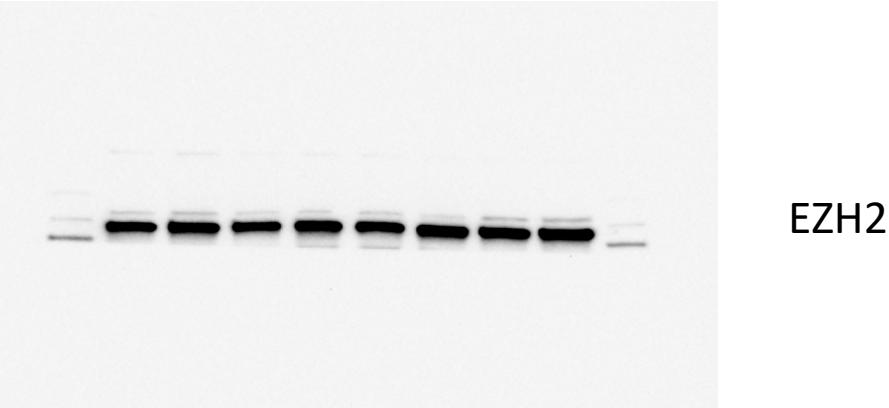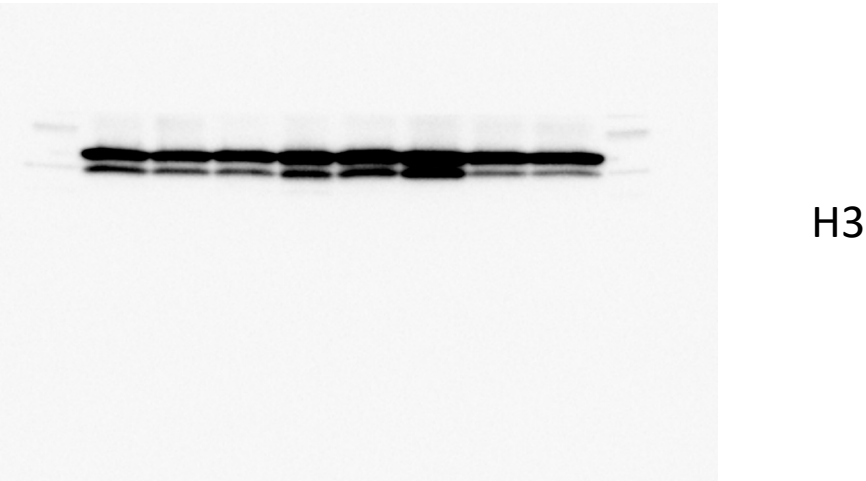

Figure 3C

KKU-055

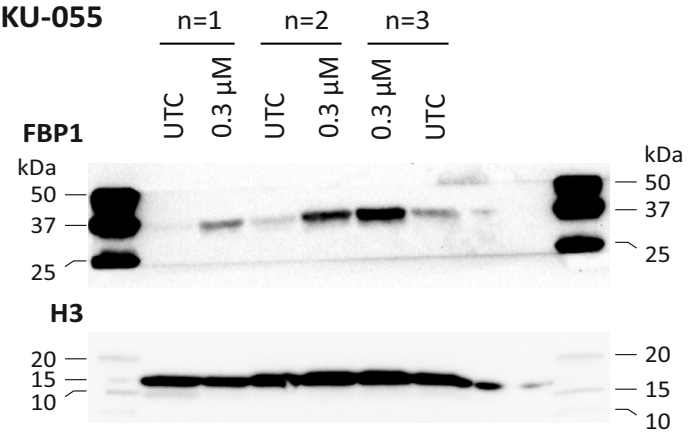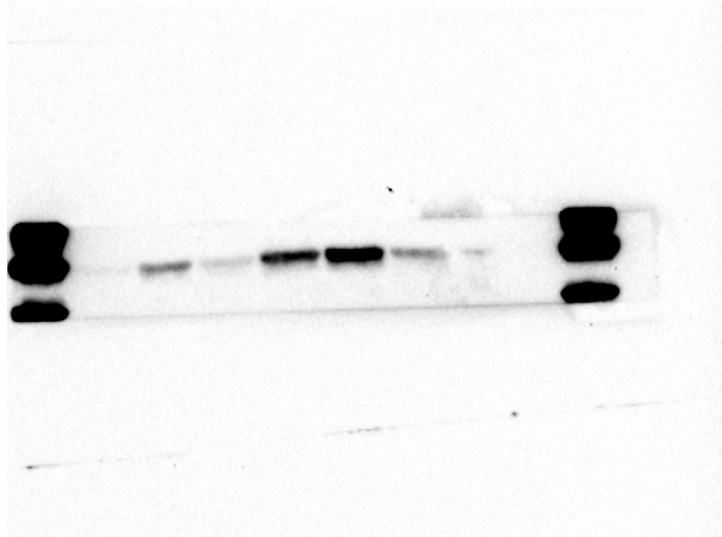

FBP1

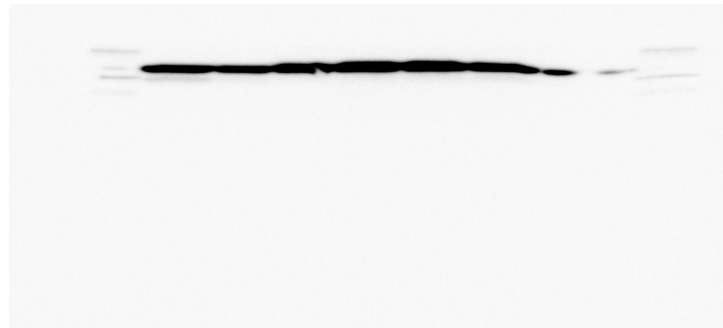

H3

NOZ

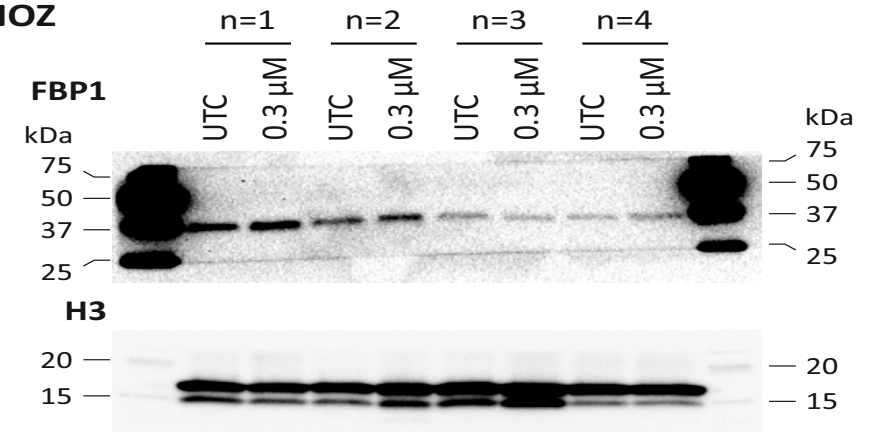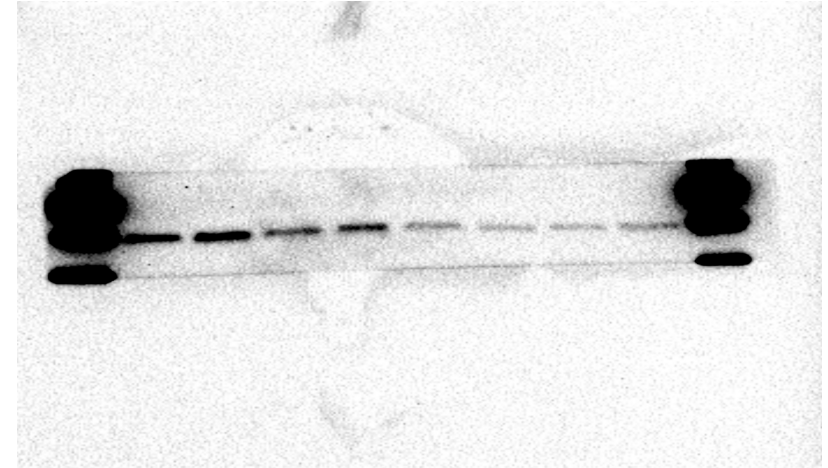

FBP1

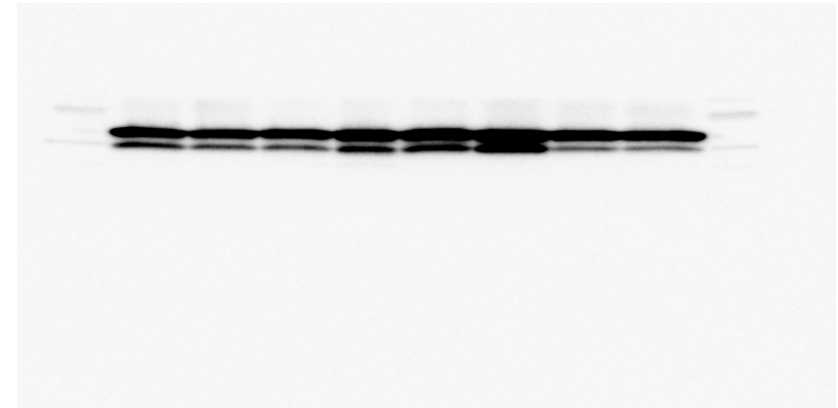

H3

Figure 4C

Supplement: Supplementary file 1 [file cancers-15-01569-s001.zip › Figure S9 Original Images for Blots.pdf]
